# Biological evaluation of isoflavonoids from *Genista halacsyi* using estrogen-target cells: Activities of glucosides compared to aglycones

**DOI:** 10.1371/journal.pone.0210247

**Published:** 2019-01-08

**Authors:** Nikolas Fokialakis, Xanthippi Alexi, Nektarios Aligiannis, Athina Boulaka, Aggeliki K. Meligova, George Lambrinidis, Eleftherios Kalpoutzakis, Harris Pratsinis, Antigoni Cheilari, Dimitra J. Mitsiou, Sofia Mitakou, Michael N. Alexis

**Affiliations:** 1 Department of Pharmacognosy and Natural Products Chemistry, Faculty of Pharmacy, National and Kapodistrian University of Athens, Athens, Greece; 2 Molecular Endocrinology Program, Institute of Biology, Medicinal Chemistry and Biotechnology, National Hellenic Research Foundation, Athens, Greece; 3 Division of Pharmaceutical Chemistry, Faculty of Pharmacy, National and Kapodistrian University of Athens, Athens, Greece; 4 Laboratory of Cell Proliferation and Ageing, Institute of Biosciences & Applications, NCSR "Demokritos", Athens, Greece; University of Naples 2, ITALY

## Abstract

The purpose of this study was to evaluate the response of estrogen target cells to a series of isoflavone glucosides and aglycones from *Genista halacsyi* Heldr. The methanolic extract of aerial parts of this plant was processed using fast centrifugal partition chromatography, resulting in isolation of four archetypal isoflavones (genistein, daidzein, isoprunetin, 8-*C-β*-D-glucopyranosyl-genistein) and ten derivatives thereof. 7-*O-β*-D-glucopyranosyl-genistein and 7,4΄-di-*O-β*-D-glucopyranosyl-genistein were among the most abundant constituents of the isolate. All fourteen, except genistein, displayed low binding affinity for estrogen receptors (ER). Models of binding to ERα could account for the low binding affinity of monoglucosides. Genistein and its glucosides displayed full efficacy in inducing alkaline phosphatase (AlkP) in Ishikawa cells, proliferation of MCF-7 cells and ER-dependent gene expression in reporter cells at low concentrations (around 0.3 μM). ICI182,780 fully antagonized these effects. The AlkP-inducing efficacy of the fourteen isoflavonoids was more strongly correlated with their transcriptional efficacy through ERα. *O*-monoglucosides displayed higher area under the dose-response curve (AUC) of AlkP response relative to the AUC of ERα-transcriptional response compared to the respective aglycones. In addition, 7-*O-β*-D-glucopyranosyl-genistein and 7,4΄-di-*O-β*-D-glucopyranosyl-genistein displayed estradiol-like efficacy in promoting differentiation of MC3T3-E1 cells to osteoblasts, while genistein was not convincingly effective in this respect. Moreover, 7,4΄-di-*O-β*-D-glucopyranosyl-genistein suppressed lipopolysaccharide-induced tumor necrosis factor mRNA expression in RAW 264.7 cells, while 7-*O-β*-D-glucopyranosyl-genistein was not convincingly effective and genistein was ineffective. However, genistein and its *O*-glucosides were ineffective in inhibiting differentiation of RAW 264.7 cells to osteoclasts and in protecting glutamate-challenged HT22 hippocampal neurons from oxidative stress-induced cell death. These findings suggest that 7-*O-β*-D-glucopyranosyl-genistein and 7,4΄-di-*O-β*-D-glucopyranosyl-genistein display higher estrogen-like and/or anti-inflammatory activity compared to the aglycone. The possibility of using preparations rich in *O-β*-D-glucopyranosides of genistein to substitute for low-dose estrogen in formulations for menopausal symptoms is discussed.

## Introduction

Isoflavonoids have a limited distribution in the plant kingdom, occurring particularly in the Papilionoideae subfamily of Fabaceae, and they are regarded as chemotaxonomic markers of *Genista L*., a large genus of spiny and non-spiny shrubs that thrive mainly around the Mediterranean [1,2]. Isoflavonoids are mostly found in plants as complex *C-β*-D- and *O-β*-D-glucosides (hereafter simplified to C- and O-glucosides) [3]. Several isoflavone aglycones are known to bind both isotypes of estrogen receptor, ERα and ERβ and often display biological activities quite similar to 17β-estradiol (estradiol) [4–6]. ERα and ERβ are co-expressed in many estrogen target cells and are known to promote and inhibit, respectively, cell proliferation as well as to regulate ER target gene transcription through ligand-induced hetero- and/or homo-dimerization, with ERα playing a dominant role in heterodimer functionality [7–9]. Dietary O-glucosides are thought to display estrogenic activity following hydrolytic release of aglycones in a manner that depends on the glucosidase activity of intestinal lumina and microflora [10–12]. In line with this notion, low nanomolar concentrations of daidzein and genistein and trace amounts of their glucosides, as compared to low micromolar levels of conjugates (mainly glucuronides and sulfates), were detected in the plasma of volunteers following consumption of various soy products [13,14]. Likewise, levels of approx. 1 μM of conjugated genistein or daidzein were detected in the plasma of individuals after ingestion of 50 mg of the respective 7-O-glucosides [15]. C-glycosylated isoflavonoids display a distribution in plants similar to that of the other isoflavonoids. Daidzein and 8-C-glycosylated daidzein, for instance, are major components of leguminous plants. They are metabolized to the highly estrogenic equol (4',7-isoflavandiol) by human intestinal microflora, with low nanomolar concentrations of total equol detected in e.g. breast tissue following ingestion of soy isoflavones [16,17]. In contrast to O-glucosides, C-glucosides cross the intestinal lumina using glucose transporters and remain metabolically stable in the circulation [12].

It has been observed that menopausal symptoms are less frequent among Asian women. This has been associated with their isoflavone-rich soy-based diet and has led to the inference that isoflavones could substitute for estrogen in treating the symptoms of menopause [18,19]. However, inconsistent clinical findings, cast doubt on the effectiveness of isoflavone-rich dietary products as alternatives to hormone therapy [18,19]. The inconsistencies could reflect the fact that isoflavones are present in plants and plant extracts mostly in the form of various complex glucosides, requiring intricate hydrolysis to release the aglycone and thus its bioavailability [11,12]. Reports on the bioavailability of isoflavones ingested in aglycone and glucoside form are conflicting. Izumi et al. [20] reported that isoflavone aglycones are absorbed faster and in greater amounts than their glucosides, while others reported that their bioavailability in glucoside form is similar [21] or even higher than their bioavailability as aglycones [22]. Isoflavone aglycones are known to cross plasma membrane by passive diffusion, predominantly depending on their hydrophobicity and solubility in water, while glucosides are highly polar entities that cannot cross plasma membrane [12]. Experiments with intestinal and non-intestinal epithelial cells have shown that O-glucosides are predominantly substrates of β-glucosidases rather than glucose transporters [23,24]. However, assessment of the biological activity of glucosides compared to aglycones using epithelial estrogen target cells yielded disparate results. For instance, Morito et al. [25] reported that genistein stimulated MCF-7 cell proliferation less than its 7-O-glucoside, although the latter was transcriptionally less active than the aglycone.

As part of our ongoing research for new phytoestrogens from Leguminous plants that form part of the Mediterranean flora [26,27], we used fast centrifugal partition chromatography (FCPC) [28], to isolate isoflavone aglycones and C- and O-glucosides from the methanolic extract of aerial parts of *Genista halacsyi* Heldr., an endemic plant of Greece. Using many different estrogen-responsive cell lines, we assessed transcriptional, estrogenic, osteoblastic, anti-osteoclastic, anti-inflammatory and neuroprotective activities of the isolated isoflavonoids. Our findings suggest that low micromolar concentrations of genistein and its *O-β*-D-glucopyranosides display similar estrogen-like activities compared to low levels of the hormone, indicating that they might substitute for low-dose estrogen therapy of menopausal symptoms.

## Materials and methods

### General

Chemicals and reagents were from Merck (Darmstadt, Germany). Evaporation of solvents was performed on a vacuum rotary evaporator (Rotavapor R-3000r, Buchi, Switzerland). FCPC was carried out on a Kromaton instrument equipped with a 1000-ml column, adjustable rotation of 200–2000 rpm and a preparative Laboratory Alliance pump with a pressure safety limit of 50 bar. NMR spectra in MeOD were recorded at 400 and 600 MHz (Bruker Advance III 600 MHz and DRX 400). 2D NMR experiments, including correlation spectroscopy (COSY), heteronuclear single-quantum correlation (HSQC) and heteronuclear multiple-bond correlation (HMBC) were performed using standard Bruker microprograms. Electrospray ionisation mass spectrometry (ESI-MS) experiments were performed on a LTQ-Orbitrap XL hybrid mass spectrometer (Thermo-Scientific, Bremen, Germany). Analytical TLC was performed on Merck Kieselgel 60 F_254_ or RP-8 F_254_ plates. Spots were visualized by UV light (254 and 365 nm) or by spraying with sulfuric vanillin. The plates were then heated for 2 min at 110°C. Preparative TLC was conducted on PLC Silica gel 60 F254 plates (1 mm). The selected zones were scraped and extracted with ethyl acetate to separate the corresponding compounds. Column chromatography was performed on silica gel 70–230 mesh (63–200 μm). Size exclusion chromatography was performed on Sephadex LH-20.

### Plant material extraction and isolation

The aerial parts of *Genista halacsyi* (Fabaceae) were collected from Mount Parnon in the Peloponnese, Greece. The plant material was identified by Dr. E. Kalpoutzakis. A Voucher specimen has been deposited in the herbarium of the Laboratory of Pharmacognosy and Natural Products Chemistry, Faculty of Pharmacy, University of Athens, Greece, under the number KL121.

Dried pulverized aerial parts of *Genista halacsyi* (1.5 kg) were extracted exhaustively by maceration using initially CH_2_Cl_2_ (3 x 2L) and then MeOH (3 x 2L). The solvents were removed under reduced pressure to give 20.1 g of a crude CH_2_Cl_2_ extract and 34.2 g of MeOH extract. The MeOH extract was submitted to fractionation using FCPC in a dual mode methodology. Fourteen solvent systems (Table A in S1 File) were selected and evaluated for their suitability for FCPC using a shaken tube test in combination with TLC. For the evaluation, a small amount of the sample was thoroughly mixed in a vial with equal volumes of the upper and lower phases of the solvent system to test and the solubility of the extract and the settling time of the biphasic system were recorded. The systems that were considered suitable were then evaluated for the distribution of the components of the extract in the two phases. Equal volumes of each phase were applied to a TLC plate and allowed to migrate in the presence of the two-phase solvent system. Optimal systems are expected to give equal distribution of the sample components between the two phases and Rf values of 0.2–0.5. This procedure showed that the biphasic system EtOAc:EtOH:H2O of 10:1:10 was the most appropriate for the fractionation of the MeOH extract of the aerial parts of *Genista halacsyi*. To proceed with FCPC, 6 L of this solvent system were prepared in a funnel and, after agitation, the phases were separated. Initially, the column (total volume of 1L) was filled with stationary (aqueous) phase using a flow rate of 20 ml/min and the revolution speed was set at 850 rpm. The extract (4.5 g) was dissolved in 30 ml of the two phases and the resulting mixture was introduced into the column, while the organic phase was passed (flow rate 10 mL/min) through the stationary phase in a tail to head or ascending mode. The effluent of the column was collected in 50 mL aliquots and after TLC analysis eight fractions (A-H) were collected. The aqueous layer was then used as mobile phase (flow rate 10 mL/min) in a head to tail or descending mode (reversed phase elution) and four aliquot-combining fractions (I-M) were collected. Fraction A (250.8 mg), containing several apolar flavonoids, as revealed by TLC analysis, was subjected to silica gel chromatography using CH_2_Cl_2_/MeOH of increasing polarity as mobile phase. Preparative TLC analysis of fractions A3 (20.1 mg) and A5 (24.8 mg), which were eluted with CH_2_Cl_2_/MeOH 98/2, afforded biochanin A (**1,** 4.7 mg) and 8-methoxy-formononetin (**2,** 8.1 mg), respectively. Furthermore, the A7 (6.9 mg) fraction, eluted with CH_2_Cl_2_/MeOH 95/5, was identified as pure genistein (**3,** 6.9 mg), while the A8 fraction (28.9 mg), eluted with CH_2_Cl_2_/MeOH 93/7, was chromatographed by preparative TLC and CH_2_Cl_2_/MeOH 90/10 as mobile phase, resulting in isolation of isoprunetin (**4**, 6.1 mg), daidzein (**5,** 2.7 mg), 3΄-methoxyisoprunetin (**6**, 2.5 mg) and 5-O-methylorobol (**7,** 3.9 mg). In addition, FCPC fractions D, F, H and K yielded directly in pure form 8-C-glucopyranosyl-genistein (**8**, 30.4 mg), 8-C-glucopyranosyl-orobol (**9**, 22.8 mg), 7-O-glucopyranosyl-isoprunetin (**10**, 24.1 mg) and 7,4΄-di-O-glucopyranosyl-genistein (**11**, 28.7 mg), respectively. Finally, 7-O-glucopyranosyl-genistein (**12**, 26.0 mg), 8-C-glucopyranosyl-3΄-O-methylorobol (**13**, 12.7 mg) and 8-C,4΄-O-diglucopyranosyl-genistein (**14**, 7.9 mg) were isolated from fractions B (152.6 mg), E (124.5 mg) and L (110.2 mg), respectively, using Sephadex column chromatography. All compounds were identified by means of spectral data (HRMS, ^1^H-NMR, ^13^C-NMR, COSY, HSQC, HMBC) and direct comparison with the respective literature data [29–33].

### Cell culture

The mouse preosteoblast cell line ‘MC3T3-E1 subclone 4’, which is capable of differentiating to mature mineralizing osteoblasts, was purchased from ATCC (ATCC CRL-2593). The cells were cultured in alpha-MEM medium (GIBCO) supplemented with 10% FBS (Biosera), 100 units/ml penicillin and 100 μg/ml streptomycin (Biochrom). Cells were subcultured before reaching confluence (approx. every 2 days). For differentiation to osteoblasts, MC3T3-E1 cells were cultured with DMEM-low glucose medium (Sigma-Aldrich) supplemented with 3% FBS, 10mM beta-glycerophosphate (Sigma-Aldrich) and 50μg/ml ascorbic acid (Sigma-Aldrich). RAW 264.7 mouse macrophages capable of differentiating to multinuclear osteoclasts were purchased from ATCC (ATCC TIB-71). The cells were maintained in alpha-MEM medium supplemented with 10% heat-inactivated ultra-low endotoxin FBS (Biosera), 100 units/ml penicillin and 100 μg/ml streptomycin. Cells were subcultured before reaching confluence (approx. every 2 days). For differentiation to osteoclasts, RAW 264.7 cells were cultured with alpha-MEM medium and 50 ng/ml Receptor Activator of Nuclear Factor kappaB ligand (RANKL, R&D Systems). ER-expressing HT22 neuronal cells were kindly provided by Dr David Schubert (The Salk Institute). The cells were maintained in Dulbecco’s Modified Eagles Medium (DMEM) supplemented with 10% FBS at a confluence not greater than 50%. MCF-7 human breast adenocarcinoma cells (from ATCC) and Ishikawa human endometrial adenocarcinoma cells (from ECACC) were cultured as recommended by the suppliers. MDA-MB-231 cells (ATCC) were cultured as already described [34]. MCF-7:D5L cells, a clone of MCF-7 cells stably transfected with an Estrogen Response Element (ERE)-endowed reporter plasmid (pERE-Gl-Luciferase), were generated and cultured as previously described [26]. HEK:ERβ cells, a clone of HEK-293 human embryonic kidney cells stably transfected with an expression plasmid coding human ERβ and an ERE-endowed reporter plasmid (pERE-tk-Luciferase), were generated and cultured as previously described [35]. Unless stated otherwise, cell culture media were from Sigma-Aldrich and FBS from Invitrogen. Dextran-coated-charcoal-treated FBS (DCC-FBS), i.e. FBS treated with 10% DCC to remove endogenous steroids, was prepared as already described [36]. The effect of test compounds on the viability of plated cells was assessed using Trypan blue as already described [34].

### Cell differentiation

Differentiation of MC3T3-E1 cells to osteoblasts has been extensively used to screen for compounds that may promote bone formation [37,38]. Differentiation of MC3T3-E1 cells was carried out in 96-well plates using 3,300 cells per well and was assessed *via* induction of, i) Alkaline Phosphatase (AlkP) activity after 6 days of treatment and, ii) mineralization of extracellular matrix after 21 days of treatment. Briefly, 24 h after plating, the cells were incubated with test compounds or vehicle i.e. the compound diluent (0.1% DMSO) and then exposed for 6 days to differentiation medium in presence or absence of differentiation factors (cf. Cell culture) with a change to fresh compounds and medium in 3 days. AlkP activity was assessed at 405 nm in a Safire II microplate reader using as substrate p-nitrophenyl-phosphate (pNPP, Sigma-Aldrich) as already described [39]. Mineralization of MC3T3 cells was assessed by staining with Alizarin red (Fluka). Cells were cultured and treated as described above for 21 days, with media and test compounds changed every 3 days, and calcium phosphate deposition was assayed as described by Gregory et al. [40]. Briefly, the cells were washed twice with PBS and fixed with 70% ethanol for 15 min on ice. The cells were stained with Alizarin red solution (40 mM, pH 4.2) for 30 min at room temperature, washed twice with distilled water and once with PBS. The dye from the stained mineral deposits was extracted with 33% acetic acid and the absorbance was measured at 405 nm using a Safire II microplate reader (Tecan).

Clones of RAW 264.7 cells competent to differentiate to multinuclear osteoclasts upon activation with RANKL provide valuable information on the regulation of osteoclast differentiation [41]. Differentiation-competent RAW cells (ATCC TIB-71) were seeded in 96-well plates at a density of 9,600 cells per well. The cells were plated in the presence of test compounds or compound diluent (0.1% DMSO) and, 4 h after plating, were exposed for 3 days to 50 ng/ml RANKL or to plain medium. Osteoclastic differentiation was assessed *via* induction of Tartrate-Resistant Acid Phosphatase (TRAP) activity. The cells were washed with PBS and incubated with 25μl lysis buffer (0.4 M NaCl, 25 mM Hepes pH 7.7, 1.5 mM MgCl_2_, 0.2 mM EDTA, 1% NP40) for 5 min on ice. Then, 25μl of assay solution (100 mM pNPP, 125 mM Sodium Acetate pH 5.2, 1mM L(+) Tartrate) were added followed by incubation at 37 ^o^C for 10 min and the absorbance was measured at 405 nm using a Safire II microplate reader.

### Neuron glutamate toxicity

Glutamate-challenged HT22 cells suffer oxidative stress-induced cell death (oxytosis) within 24 h due to glutathione depletion and consequent massive accumulation of ROS [42]. The efficacy of test compounds to prevent oxytosis of HT22 cells was assessed as already described [43]. Briefly, HT22 cells were plated in 96-well flat bottom plates at a density of 4,000 cells per well in 100 μl of DMEM (low glucose) containing 2% FBS. 24 h after plating, the cells were treated with test compounds or compound diluent (0.1% DMSO) and then challenged with 5 mM glutamate for 24 h. Relative numbers of viable cells were determined following conversion of 3-(4,5-dimethylthiazol-2-yl)-2,5-diphenyl-tetrazolium bromide (MTT, Sigma-Aldrich) to coloured formazan, with the difference in optical density at 550 and 690 nm taken as a measure of viable cell number. Non-challenged cells served to assess test compound effects on cell viability, whereas challenged cells served to assess the neuroprotective activity of test compounds.

### Cell proliferation

Test compound effects on the proliferation of MCF-7 and MDA-MB-231 cells were assessed as previously described [34]. Briefly, the cells were plated in 96-flat-bottom-well plates at a density of 8,000 cells per well in phenol-red-free MEM supplemented with 1 μg/mL insulin and 5% DCC-FBS. 24 h after plating, the cells were exposed for 72 h to 0.1 nM estradiol (Sigma-Aldrich), test compounds or vehicle (≤ 0.2% DMSO), in the absence or presence of 1 μM of the ER-degrader ICI182,780 (Tocris Bioscience), and viable cells were determined using MTT as described above. The difference in optical density at 550 and 690 nm was taken as a measure of the number of viable cells and used to derive the number of compound-treated cells relative to that of vehicle-treated cells. Relative numbers of cells exposed to estradiol or to ICI182,780 acted as positive and negative control, respectively.

### Estrogen receptor activities

#### Transcriptional activity

Assessment of ER/ERE-dependent luciferase gene transcription in MCF-7:D5L (express ERα) and HEK:ERβ cells (express ERβ) and determination of ER transcriptional agonism was carried as already described [44]. Briefly, the cells were plated in 96-well plates at a density of 12,000 cells per well in phenol-red-free MEM (MCF-7:D5L cells) or phenol-red-free DMEM (HEK:ERβ cells) supplemented with 1 μg/mL insulin and 5% DCC-FBS. Three days after plating, the cells were exposed for 18 h to 0.1 nM estradiol, to the indicated concentrations of test compounds or to vehicle (≤0.2% DMSO), in the absence or presence of 1 μM ICI182,780, and luciferase expression was assessed in a Safire II microplate reader (Tecan) using the Steady-Glo Luciferase Assay System (Promega). Cells exposed to vehicle, estradiol and/or ICI182,780 served as controls. Dose-response data of ERα/ERE-dependent luciferase expression were curve fitted using SigmaPlot10 (SPSS Inc) and the area under the dose-response curve (AUC) was calculated using >150 trapezoids and Microsoft Excel. The AUC of luciferase response of **3**, **4**, **5** and **8** (taken as the archetypal isoflavones) was calculated up to the concentration that provided a response most comparable to that of 0.1 nM estradiol (i.e. up to 0.3, 10, 1 and 3 μM for **3**, **4**, **5** and **8**, respectively). The concentration of **3** (i.e. 0.3 μM) that mounted an estradiol-like luciferase response was used to calculate the AUC of **1**, **11** and **12**, while that of **4** (i.e. 10 μM) was used to calculate the AUC of **6**, **7** and **10**, that of **5** (i.e. 1 μM) was used for the AUC of **2,** and that of **8** (i.e. 3 μM) was used for the AUC of **9**, **13** and **14**. The AUC of the isoflavonoids of each group were then expressed as % of the AUC of the respective archetypal isoflavone. Since AUC data combine the efficacy as well as the potency of cell response into a single parameter, the %AUC data were considered here as a more reliable measure of relative ER/ERE-dependent luciferase expression of the test compounds.

#### AlkP expression

Induction of AlkP expression of Ishikawa cells is considered as a reliable in vitro measure of estrogenic activity [45,46]. Assessment of AlkP expression in Ishikawa cells was carried as already described [26]. Briefly, the cells were plated in 96-well plates at a density of 12,000 cells per well in phenol-red-free MEM supplemented with 1 μg/mL insulin and 5% DCC-FBS. 24 h after plating, the cells were exposed for 72 h to 0.1 nM estradiol, to the indicated concentrations of test compounds or to vehicle (≤ 0.2% DMSO), in the absence or presence of 1 μM ICI182,780, and AlkP activity was assessed at 405 nm in a Safire II microplate reader using pNPP. Cells exposed to vehicle, estradiol and/or ICI182,780 served as controls. Data on the dependence of AlkP activity on isoflavonoid concentration were curve fitted using SigmaPlot10 (SPSS Inc) and the numerical data of curve fitting were used to determine AUC as described above, using for the calculation of AUC of a particular AlkP response the very same concentration used for the respective luciferase response. The AUC of AlkP responses of the isoflavonoids of a group were expressed as % of the AUC of the luciferase response of the respective archetypal isoflavone.

#### ER binding

Test compound affinity of binding to ERα and ERβ relative to that of estradiol (relative binding affinity, RBA) was assessed using competitor assay kits with full-length recombinant ERα and ERβ (Invitrogen) as already described [26]. Briefly, the concentration of 17β-estradiol or test compound that inhibited the binding of the fluorescent estrogen ES2 (Invitrogen) to ERα or ERβ by 50% (IC_50_) was used to calculate RBAα, RBAβ and the ERβ-binding selectivity as already described [26].

### TNFα mRNA expression

To assess TNFα mRNA expression, RAW 264.7 cells were plated in 6-well plates in alpha-MEM medium supplemented with 10% DCC-FBS, at a density of 300,000 cells per well, maintained in culture for 72 h and then exposed to vehicle (0.3% DMSO) or 3 μΜ test compound for 20 min or 24 h prior to stimulation with 100 ng/ml lipopolysaccharide (Sigma L4391) for 1 h. Extraction of total RNA, reverse transcription (RT) and quantitative PCR (qPCR) analysis of mRNA expression levels were performed as previously described [47]. Relative gene expression levels were calculated by the comparative Ct method using the formula 2^(-ΔCt)^. Tumor necrosis factor alpha (TNFα) mRNA levels were normalized to the respective levels of glycerol-3-phosphate dehydrogenase (GAPDH). Test compound effects on the relative number of viable cells were assessed using crystal violet and a Safire II microplate reader (Tecan) as previously described [39]. The difference in optical density at 550 and 690 nm was taken to measure the actual number of viable cells. The following primers were used:

Mouse TNFα:      FW: 5’-TCTCATTCCTGCTTGTGGCA-3’

                            RV: 5’-AGGGTCTGGGCCATAGAACT-3’

Mouse GAPDH:  FW: 5’-CATGGC CTTCCGTGTTCCTA-3’

                            RV: 5’-CCTGCTTCACCACCTTCTTGAT-3’

### Molecular modeling

#### LogP values

Theoretical logP values were calculated using QikProp software as implemented on Maestro 10. (Schrödinger Release 2017–4: QikProp, Schrödinger, LLC, New York, NY, 2017)

#### Receptor and ligand preparation

PDB entries 1X7R [48] and 2P15 [49] were used as starting structures for the ligand-binding domain of ERα having H12 in agonist position, in complex with genistein and ortho-trifluoromethyl-phenylvinyl estradiol (EZT) respectively. The protein preparation wizard was utilized as implemented on Maestro Software. All crystallographic water molecules were deleted except the one between ARG 394 and GLU 353. HIS 524 was protonated on NE2. All ligands were designed using Maestro 10. (Schrödinger Release, 2017–4: Protein Preparation Wizard; Epik, Schrödinger, LLC, New York, NY, 2016; Impact, Schrödinger, LLC, New York, NY, 2016; Prime, Schrödinger, LLC, New York, NY, 2017. LigPrep, Schrödinger, LLC, New York, NY, 2017).

#### Induced fit docking calculations

Schrödinger developed and validated an Induced Fit Docking (IFD) protocol based on Glide and the Refinement module in Prime for accurate prediction of ligand binding modes and concomitant structural changes in the receptor. (Schrödinger Release, 2017–4: Schrödinger Suite, 2017–4 Induced Fit Docking protocol; Glide, Schrödinger, LLC, New York, NY, 2016; Prime, Schrödinger, LLC, New York, NY, 2017) [50]. The IFD protocol models induced fit docking of ligands using the following steps:

1 An optional constrained minimization of the receptor (protein preparation, refinement only) with an RMSD cutoff of 0.18 Å. Normally, this is done when preparing the protein with the Protein Preparation Wizard.
2 Initial Glide docking of each ligand using a softened potential (van der Waals radii scaling), and optional removal of side chains and application of constraints. By default, a maximum 20 poses per ligand are retained.
3 Prime side-chain prediction for each protein-ligand complex, on residues within a given distance of any ligand pose (default 5 Å), with optional inclusion or exclusion of other residues, and an optional implicit membrane model. Prime minimization of the same set of residues and the ligand for each protein-ligand complex pose. The receptor structure in each pose reflects an induced fit to the ligand structure and conformation.
4 Glide redocking of each protein-ligand complex structure within a specified energy of the lowest-energy structure (default 30 kcal/mol). The ligand is rigorously docked, using default Glide settings, into the induced-fit receptor structure.
6 Estimation of the binding energy (IFDScore) for each output pose.

### Statistical analysis

Data were expressed as mean ± standard error of the mean (SEM) of at least three independent experiments carried out in triplicate. Statistically significant differences were determined using one-way ANOVA and SPSS 13.0 software unless stated that *t-*test was used. Differences were considered significant for values of *p* ≤ 0.05.

## Results

### Isolation of isoflavones and isoflavone glucosides

In continuation of a screening programme investigating plants of the Mediterranean basin for compounds with estrogen-like activity, we observed that the methanolic extract of the aerial parts of *Genista halacsyi* displayed considerable AlkP-inducing activity in Ishikawa cells. In order to fractionate the extract rapidly and effectively, we used FCPC. Initially, fourteen solvents systems (Table A in S1 File) were evaluated for the solubility of the extract, the settling time of the biphasic system (should be shorter than 30 seconds) and the distribution of the components in the two phases by TLC. The biphasic systems 9, 11, 13 and 14 were rejected due to the formation of emulsion. On the other hand, TLC revealed an inappropriate partition of the ingredients in two phases in the case of systems 1–7, 10 and 12. Finally, the biphasic system EtOAc : EtOH : H_2_O (10 : 1 : 10) was selected as the most suitable. Fractionation of 4.5 g of the extract using 2 L of organic solvents yielded in 4 h several fractions of 4.4 g total weight amounting to 98.2% recovery. The FCPC separation process yielded directly in pure form four isoflavone glucosides (**8–11**). In addition, this process, in combination with Sephadex column chromatography, provided three more isoflavone glucosides (**12**–**14**) and, in combination with preparative TLC, seven aglycones (**1**–**7**).

**Fig 1:**
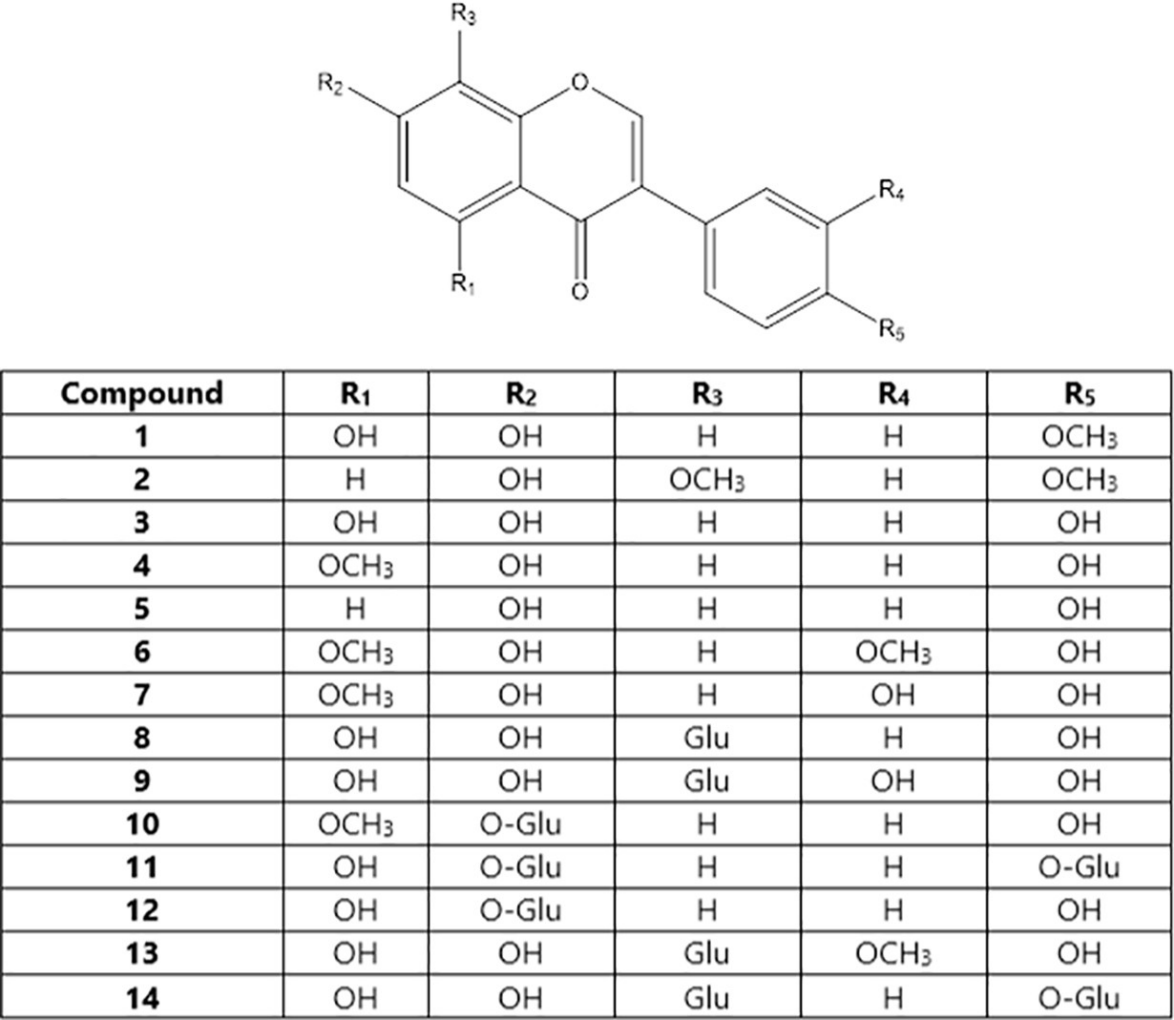
Isoflavonoids isolated from *Genista halacsyi*. Biochanin A (**1**), 8-methoxyformononetin (**2**), genistein (**3**), isoprunetin (**4**), daidzein (**5**), 3΄-methoxyisoprunetin (**6**), 5-O-methylorobol (**7**), 8-C-glucopyranosylgenistein (**8**), 8-C-glucopyranosyl-orobol (**9**), 7-O-glucopyranosyl-isoprunetin (**10**), 7,4΄-di-O-glucopyranosylgenistein (**11**), 7-O*-*β-D-glucopyranosyl-genistein (**12**), 8-C-glucopyranosyl-3΄-O-methylorobol (**13**) and 8-C,4΄-O-diglucopyranosyl-genistein (**14**).

The structure of the isolated components (Fig 1) was elucidated by High Resolution Mass Spectrometry and 1D and 2D NMR spectroscopy, and by comparison with literature data (Tables B-O in S1 File). The quantities of pure isoflavonoids isolated from 4.5 g of extract ranged from 2.5 (**6**) to 30.4 mg (**8**). The total amount of genistein (6.1 mg) and its 7-O- and 7,4΄-di-O-glucosides (28.7 and 26.0 mg, respectively) amounted to 33% of the total amount of isolated isoflavonoids. For the ensuing biological evaluation, the isoflavonoids were taken to belong to 4 groups, each comprising an archetypal isoflavone (underlined) and members deriving from minimal addition(s) to the core structure of the archetypal entity (underlined): the genistein group (**1**, **3**, **11**, **12**), the isoprunetin (5-methyl-genistein) group (**4**, **6**, **7**, **10**), the daidzein group (**2**, **5**) and the 8-C-glucopyranosyl-genistein group (**8**, **9**, **13**, **14**).

### Relative binding affinities for ERα and ERβ

Initially we determined the binding affinity relative to estradiol (RBA) of isoflavonoids for ERα (RBAα) and ERβ (RBAβ). Table 1 shows that, with the RBAα and RBAβ of estradiol set equal to 100, the RBA of isoflavonoids ranged from 68.4 (RBAβ of **3**) to less than 0.01 (RBAα and RBAβ of **11**). Specifically, none of the 14 isoflavonoids displayed RBAα>1, six displayed values between 0.1–1 (**1**, **3**, **6, 7**, **9**, **13**) and eight displayed values <0.1 (**2**, **4**, **5**, **8**, **10**–**12**, **14**). Similarly, one isoflavonoid displayed RBAβ>>1 (**3**), seven displayed values between 0.1–1 (**1, 2, 4**–**7, 13**) and six displayed values <0.1 (**8**–**12, 14**). Notably, **3** displayed high selectivity for ERβ, **1** and **4**–**6** displayed moderate selectivity for ERβ, while none of the 14 isoflavonoids displayed appreciable selectivity for ERα. It is evident that the absence of a 5-OH group in **5**, the substitution of a methoxy group for the 5-OH in **4** and the 8-C-glycosylation in **8** rendered all three of them much less ERβ-selective and much weaker ER binders compared to **3**. It is also evident that the RBA of archetypal isoflavones ranked differently compared to other members of their group: **3** displayed higher RBAβ compared to **1**, but **4** displayed lower RBAα compared to **6** and **7** and this was also the case with the RBAβ of **4** compared to **6**.

**Table 1:** Relative ER-binding affinity (RBA) and selectivity^a^.

Compound	RBAα	RBAβ	RBAβ / RBAα
Estradiol	100	100	1.00
Genistein group			
**3**	0.59 ± 0.21	68.4 ± 11.1	116
**1** ^b^	0.24 ± 0.11	0.92 ± 0.16	3.83
**11**	<0.01	<0.01	(-)
**12**	0.04 ± 0.03	0.07 ± 0.02	1.75
Isoprunetin group			
**4**	0.03 ± 0.01	0.15 ± 0.05	5.00
**6**	0.14 ± 0.04	0.63 ± 0.14	4.50
**7**	0.13 ± 0.04	0.15 ± 0.04	1.15
**10**	0.02 ± 0.01	0.03 ± 0.01	1.50
Daidzein group			
**5** ^c^	0.04 ± 0.02	0.36 ± 0.09	9.00
**2**	0.06 ± 0.02	0.15 ± 0.04	2.50
8-C-glucopyranosyl-genistein group			
**8**	0.05 ± 0.01	0.09 ± 0.02	1.80
**9**	0.11 ± 0.03	0.05 ± 0.01	0.45
**13**	0.12 ± 0.05	0.12 ± 0.04	1.00
**14**	0.02 ± 0.01	0.05 ± 0.02	2.50

^a^ RBA values (mean ± SEM of three independent experiments) of the test compounds for ERα (RBAα) and ERβ (RBAβ) were calculated by [RBA = (IC_50 estradiol_/IC_50 compound_) × 100]; IC_50_ values are estradiol or test compound concentrations that inhibit binding of fluorescent estrogen ES2 (1 nM) to ERα and ERβ by 50%. RBAα and RBAβ of estradiol were set equal to 100. Selectivity for ERβ was classified as high, moderate or weak depending on whether RBAβ/RBAα was >10, 3–10 or <3, respectively.

^b^ data from Fokialakis et al. [51].

^c^ data from Halabalaki et al. [27].

We calculated the mean value (and the range) of previously reported RBAα and RBAβ values to be, respectively, 1.51 (0.03–4) and 20.7 (0.86–87) for **3**, 0.20 (0.01–0.55) and 0.65 (0.04–2.11) for **5**, 0.18 (0.02–0.34) and 0.69 (0.18–1.20) for **1**, and <0.01 and <0.01 for **12** [4,25,52], in good agreement with the data of Table 1.

### Models of binding to ERα

**Fig 2:**
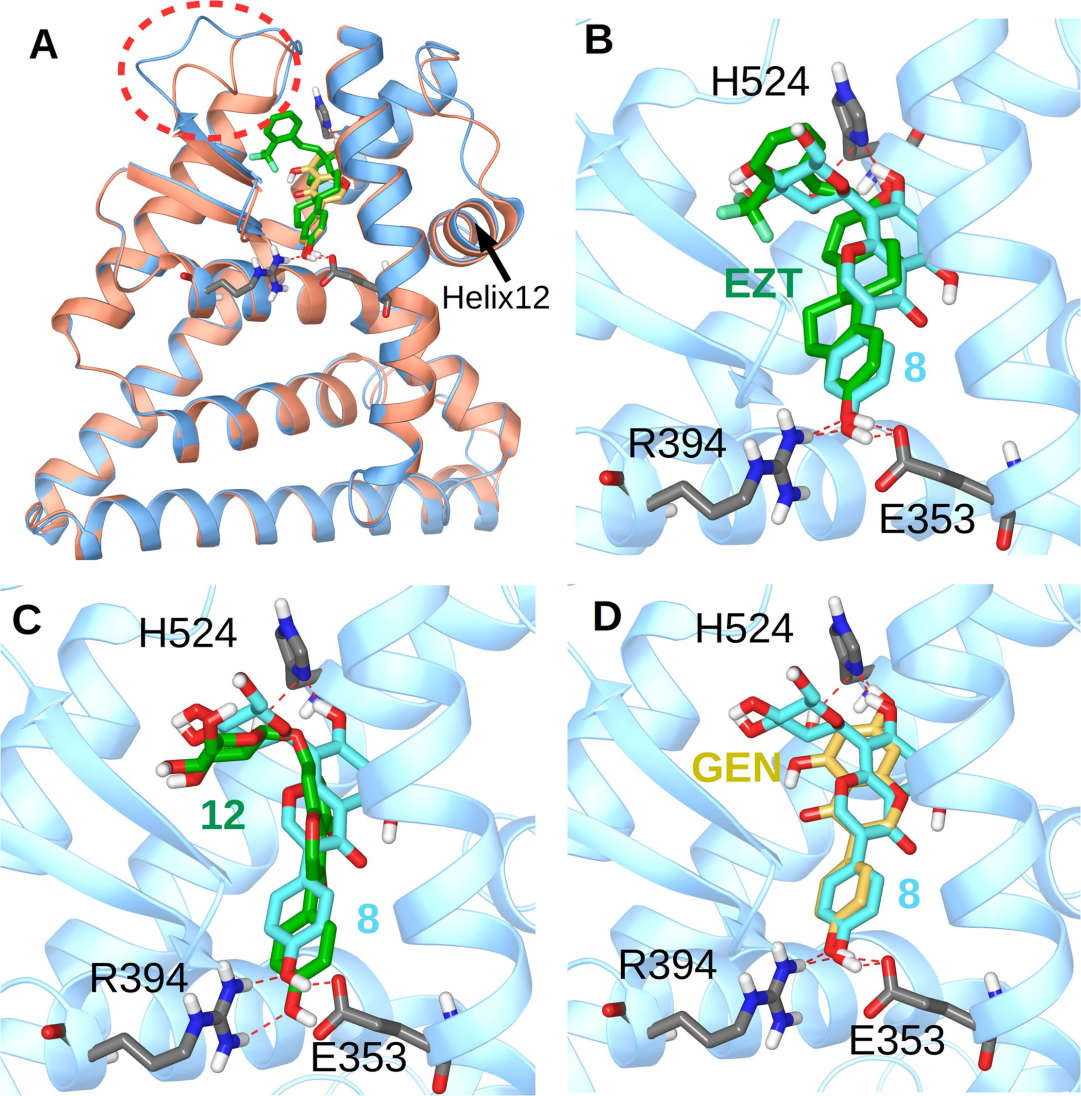
(A) Ribbon representation of ERα in complex with genistein (GEN, orange ribbons) and ortho-trifluoromethylphenylvinyl estradiol (EZT, light blue ribbons). The flexible region which accommodates the bulky group of EZT is inside the red circle. (B) Superposition of crystal structure of EZT with global minimum structure of **8** inside the binding pocket of ERα. (C) Superposition of global minimum structure of **12** with global minimum structure of **8** inside the binding pocket of ERα. (D) Superposition of crystal structure of genistein with global minimum structure of **8** inside the binding pocket of ERα. For clarity reasons Helix 1 has been removed from all figures. Hydrogen bonds are depicted with red dashed lines.

To better understand the binding affinity of the 14 isoflavonoids, we run docking calculations and we analyzed the physicochemical properties as described by their logP values. For docking calculations two different structures of ERα were utilized, having helix-12 (H12) of ERα in the agonist position, one in complex with genistein (PDB entry 1X7R) [48] and the other in complex with ortho-trifluoromethyl-phenylvinyl estradiol (EZT, PDB entry 2P15) [49]. The latter is a very good example where the plasticity of the receptor allows fitting of a relatively voluminous estradiol analog (Fig 2A). The binding pocket of ERα has a hydrophobic binding cavity, with only two hydrophilic spots, R394/E353 and H524 in a distance of 12Å. Glucosides **8**–**14** displayed very low logP values (Table P in S1 File), reflecting their hydrophilic profile which perturbs binding to ERα and lowers binding affinity for the receptor.

Upon Induced Fit Docking, all compounds except **11** and **14** fitted inside the binding pocket of 2P15 ligand binding domain. However, only the aglycons (**1**–**7**) were able to bind on 1X7R, while the remainder isoflavonoids were rejected because of their volume. Compounds **11** and **14** contain two glucose residues, of which one is at position 4’, and are the most hydrophilic of the fourteen isoflavonoids (Table P in S1 File). Compound **8** bound to ERα very similarly to EZT and genistein (Fig 2B and 2D). However, although **12** was able to bind to ERα with similar orientation (Fig 2C), the inability to form a hydrogen bond with H524 due to the absence of a OH group at position 7 increased the theoretical free energy of binding by 1.2 kcal/mol compared to compound **8**.

### Agonism of gene transcription, AlkP expression and cell proliferation

**Fig 3:**
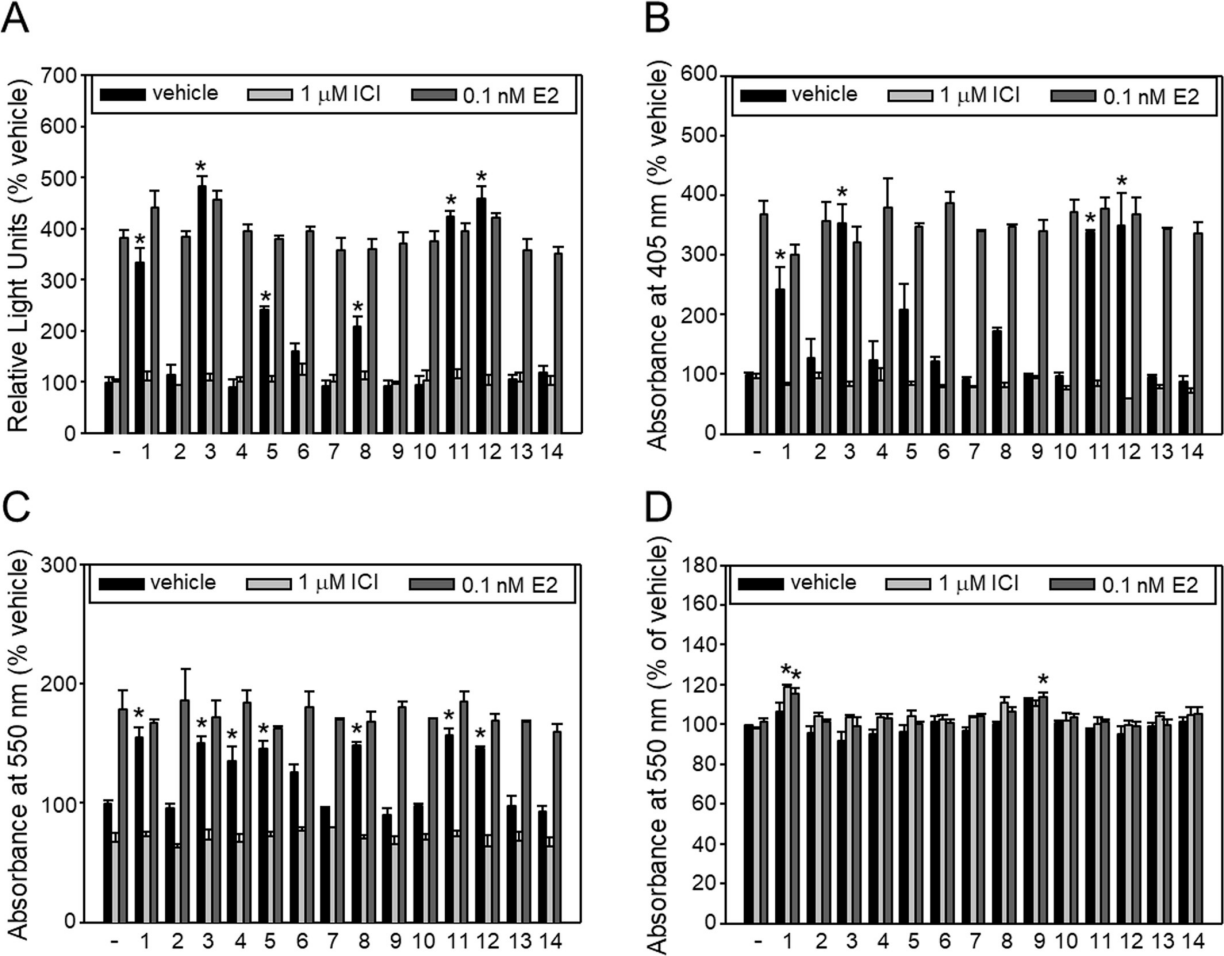
Effect of isoflavonoids on ERE-dependent gene transcription, alkaline phosphatase expression and breast cancer cell proliferation. (A) Relative activity of ERE-dependent luciferase gene transcription in estrogen-free MCF-7:D5L cells, (B) Relative AlkP expression of estrogen-free Ishikawa cells and (B) relative number of viable estrogen-free (C) MCF-7 cells and (D) MDA-MD-231 cells following incubation with vehicle or isoflavonoids (1 μM) in the absence or presence of either 1 μM ICI182,780 or 0.1 nM estradiol. AlkP expression was assessed at 405 nm through the hydrolysis of p-nitrophenyl phosphate to coloured p-nitrophenol. Viable cell numbers were assessed at 550 nm through the conversion of MTT to coloured formazan. Absorbance in the presence of vehicle was set equal to 100. Data are Mean±SEM from at least three independent experiments carried out in triplicate. *, *p<*0.05 *vs* no treatment (ANOVA).

We then examined whether the isoflavonoids induce, i) ERE-dependent luciferase gene transcription in MCF-7:D5L cells (known to express ERα), ii) expression of AlkP in Ishikawa endometrial adenocarcinoma cells (known to express ERα and ERβ), iii) stimulate proliferation of MCF-7 breast adenocarcinoma cells (known to express ERα but not ERβ) and, iv) affect proliferation of MDA-MB-231 breast adenocarcinoma cells (known to express neither ERα nor ERβ). We also examined whether ICI182,780, a full antagonist of ER, suppresses modulation of these activities by the 14 isoflavonoids and whether the isoflavonoids suppress induction of these activities by 0.1 nM estradiol (postmenopausal level of the hormone). The isoflavonoids were tested at 1 μM, since higher concentrations are hardly attainable in vivo and hence of little practical interest. Fig 3A shows that luciferase gene transcription was induced 3.36-fold in the presence of 0.1 nM estradiol but not in the presence of 1 μM ICI182,780; that **3**, **11** and **12** induced gene transcription to levels very similar to estradiol; that isoflavonoid-induced gene transcription was fully suppressed by ICI182,780; and that none of the isoflavonoids was able to suppress estradiol-induced gene transcription.

Fig 3B shows that AlkP expression was induced 3.68-fold in the presence of 0.1 nM estradiol but not in the presence of 1 μM ICI182,780; that **3**, **11** and **12** induced AlkP expression to levels very similar to estradiol; that isoflavonoid-induced AlkP expression was fully suppressed by ICI182,780; and that none of the isoflavonoids was able to suppress estradiol-induced AlkP expression. Some isoflavonoids (e.g. **12**) displayed somewhat lower AlkP expression compared to vehicle in the presence of ICI182,780; however, these effects were not statistically significant. Fig 3C shows that proliferation of MCF-7 cells was induced 1.80-fold in the presence of 0.1 nM estradiol but not in the presence of 1 μM ICI182,780; that several isoflavonoids induced cell proliferation to levels similar or somewhat lower to estradiol; that all these cell proliferation effects were fully suppressed by ICI182,780; and that none of the isoflavonoids was able to suppress estradiol-induced cell proliferation. Finally, Fig 3D shows that the proliferation of MDA-MB-231 cells was not affected by estradiol, ICI182,780 or any of the isoflavonoids, with the exception of few very minor stimulatory effects which are considered haphazard.

The data of Fig 3 were used to deduce estrogen agonist effects of isoflavonoids at 1 μΜ as compared to the respective agonist effect of 0.1 nM estradiol. Regarding induction of AlkP, only four isoflavonoids displayed statistically significant agonist effects that were similar to (**3**, **11**, **12**) or lower than (**1**) the effect of estradiol (Table 2, column 2).

**Table 2:** Agonism of AlkP expression, cell proliferation and gene transcription.

Compound	Induction of AlkP expression (Ishikawa cells)	Induction of cell proliferation (MCF-7 cells)	Induction of luciferase gene transcription through ERα or ERβ	
			MCF-7:D5L cells	HEK:ERβ cells
	Agonism^a^ at 1μΜ (% of 0.1nM E2)	Agonism^a^ at 1μΜ (% of 0.1nM E2)	Agonism^a^ at 1μΜ (% of 0.1nM E2)	Agonism^a^ at 1μΜ (% of 0.1nM E2)
Estradiol	100 ± 9 #	100 ± 19 #	100 ± 5 #	100 ± 12 #
vehicle	0 ± 1 *	0 ± 4 *	0 ± 4 *	0 ± 5 *
Genistein group				
**3**	110 ± 11 (Full) #	79 ± 6 (Full) #	135 ± 7 (Full) # *	116 ± 18 (Full) #
**1**	53 ± 9 (Partial) # *	70 ± 10 (Full) #	83 ± 16 (Full) #	31 ± 15 (Weak) *
**11**	105 ± 9 (Full) #	79 ± 5 (Full) #	114 ± 4 (Full) #	80 ± 20 (Full) #
**12**	101 ± 5 (Full) #	79 ± 9 (Full) #	127 ± 8 (Full) # *	121 ± 18 (Full) #
Isoprunetin group				
**4**	Marginal	19 ± 6 (Weak) # *	Marginal	27 ± 5 (Weak) # *
**6**	Marginal	32 ± 3 (Weak) # *	22 ± 5 (Weak) # *	81 ± 5 (Full) #
**7**	Marginal	Marginal	Marginal	13 ± 4 (Weak) *
**10**	Marginal	Marginal	Marginal	Marginal
Daidzein group				
**5**	54 ± 4 (Partial) # *	59 ± 7 (Partial) #	50 ± 12 (Partial) # *	84 ± 8 (Full) #
**2**	Marginal	Marginal	Marginal	Marginal
8-C-glucopyranosyl-genistein group				
**8**	21 ± 5 (Weak) # *	44 ± 2 (Partial) #	39 ± 7 (Partial) # *	46 ± 3 (Partial) # *
**9**	Marginal	Marginal	Marginal	Marginal
**13**	15 ± 4 (Weak) # *	Marginal	Marginal	Marginal
**14**	Marginal	Marginal	Marginal	18 ± 1 (Weak) # *

^a^ Agonism was calculated by: [(Effect test compound—Effect _vehicle_) × 100 / (Effect _0.1nM E2_—Effect _vehicle_)] Agonism was classified as full, partial, weak or marginal depending on whether induction of the effect was, respectively, ≥67, 34–66, 11–33 or ⩽10% of the respective agonism of 0.1 nM estradiol

*, *p<*0.05 *vs* Agonism of 0.1 nM estradiol (*t*-test)

#, *p<*0.05 *vs* no treatment (*t*-test)

E2 = estradiol

With reference to induction of MCF-7 cell proliferation, eight isoflavonoids displayed statistically significant agonist effects that were similar to (**1**, **3**, **5**, **8**, **11**, **12**) or lower than (**4**, **6**) the effect of estradiol (Table 2, column 3). With respect to ERα-dependent induction of gene transcription, seven isoflavonoids displayed statistically significant agonist effects that were higher than (**3**, **12**), similar to (**1**, **11**) or lower than (**5**, **6**, **8**) the effect of estradiol (Table 2, column 4). Finally, in relation to ERβ-dependent induction of gene transcription, nine isoflavonoids displayed statistically significant agonist effects that were similar to (**3**, **6**, **11**, **12**) or lower than (**1**,**4**, **7**, **8**, **14**) the effect of estradiol (Table 2, column 5). Agonist effects higher than or similar to the respective effect of estradiol were mostly classified as full, effects lower than the effect of estradiol were classified as partial or weak, while effects that were not statistically significant were classified as marginal (Table 2). Interestingly, the data of Table 2 revealed that AlkP-inducing agonisms were more strongly correlated with transcriptional agonisms through ERα (Pearson’s *r* = 0.945; 2-tailed *p* = 0.000; n = 14) than through ERβ (Pearson’s *r* = 0.734; 2-tailed *p* = 0.004; n = 13).

### Dose-response variations of AlkP-inducing and ERα-transcriptional activities

**Fig 4:**
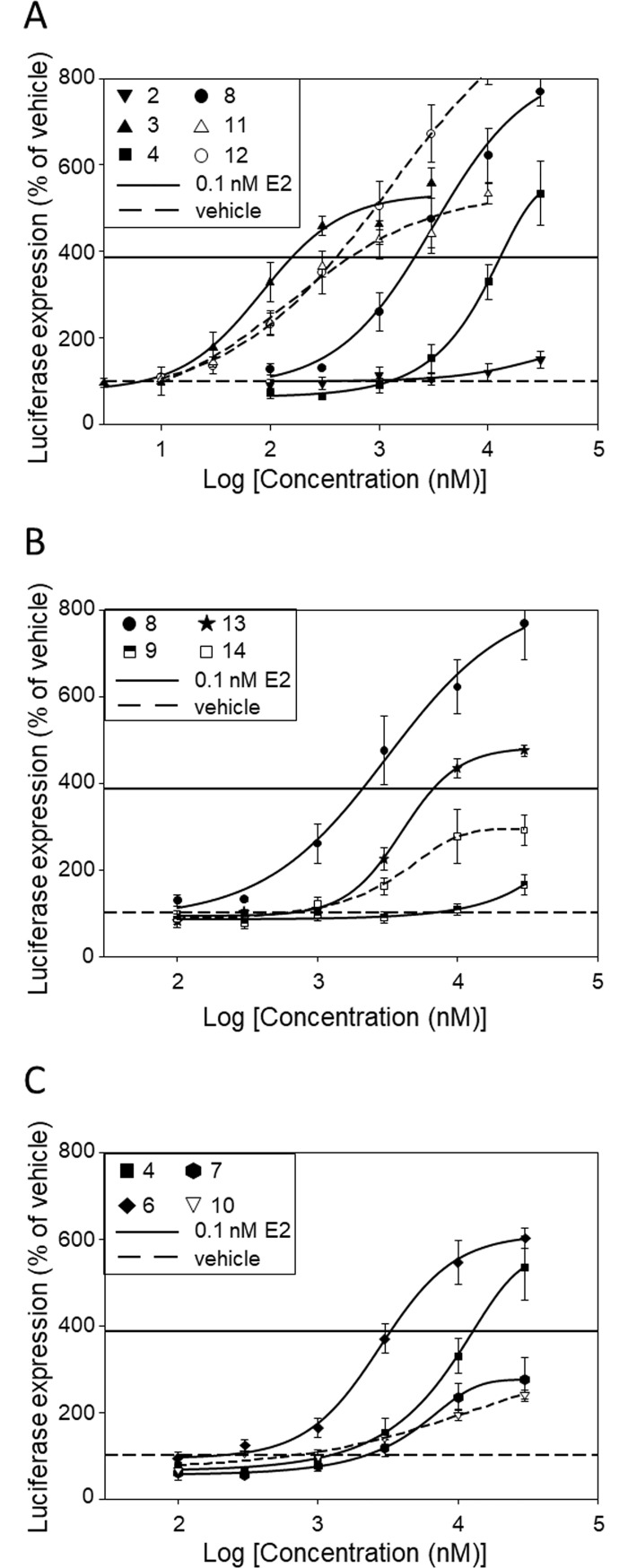
Effect of isoflavonoids on ERE-dependent luciferase expression. Luciferase expression of estrogen-free MCF-7:D5L cells following incubation with increasing concentrations of isoflavonoids **2**–**4**, **8**, **11**, **12** (A), **8**, **9**, **13**, **14** (B) and **4**, **6**, **7**, **10** (C). Expression in the presence of vehicle was set equal to 100. Basal expression in the presence of vehicle is shown by a dashed line while expression in the presence of 0.1 nM estradiol is shown by a straight line. Values (% of vehicle) are mean±SEM of an experiment carried out in triplicate. ERE, estrogen response element.

**Fig 5:**
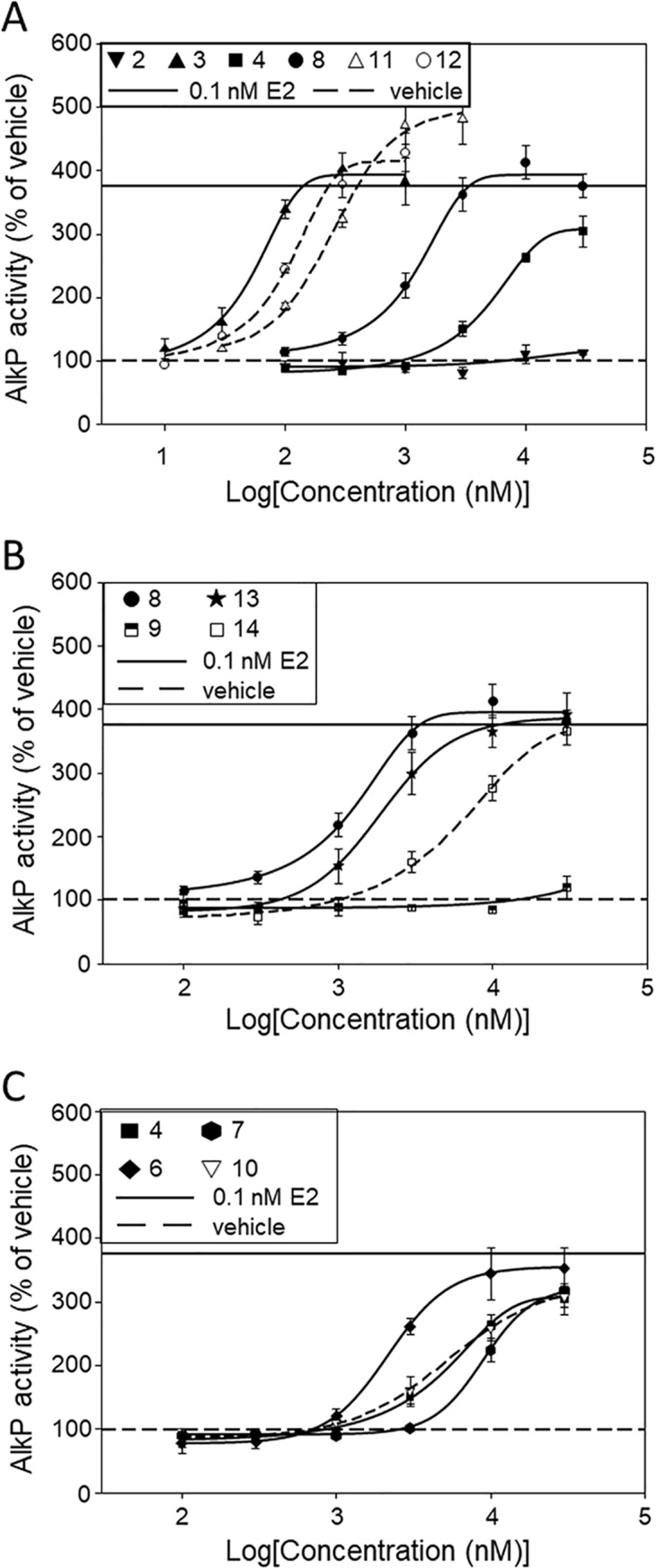
Effect of isoflavonoids on alkaline phosphatase expression. Alkaline phosphatase (AlkP) expression of estrogen-free Ishikawa cells following incubation with increasing concentrations isoflavonoids **2**–**4**, **8**, **11**, **12** (A), **8**, **9**, **13**, **14** (B) and **4**, **6**, **7**, **10** (C). Expression in the presence of vehicle was set equal to 100. Basal expression in the presence of vehicle is shown by a dashed line while expression in the presence of 0.1 nM estradiol is shown by a straight line. Values (% of vehicle) are mean±SEM of an experiment carried out in triplicate.

Having shown that the agonist effects of the O-glucosides of genistein rivaled those of the aglycone when tested at 1 μM, we set out to investigate in more detail how glucose moieties and other substituents in the structure of genistein affect the AlkP-inducing response compared to the ERα-transcriptional response in a dose-dependent manner. Figs 4A and 5A show that luciferase and AlkP responses of **4** and **8** were considerably weaker compared to **3**, while those of **11** and **12** were fairly comparable to **3**. Similarly, Figs 4B and 5B show that luciferase and AlkP responses of **9**, **13** and **14** were weaker compared to **8** and Figs 4C and 5C show that luciferase and AlkP responses of **4**, **7** and **10** were considerably weaker compared to **6**.

Table 3 shows IC_25_ (columns 2 and 5) and IC_50_ data (columns 3 and 6) from several experiments such as those shown in Figs 4 and 5 as well as AUC data (columns 4 and 7), as normalized on a group basis using the AUC of ERα/ERE-dependent luciferase response of **3** (genistein group), **4** (isoprunetin group), **5** (daidzein group) and **8** (8-C-glucopyranosyl-genistein group) up to 0.3, 10, 1.0 and 3.0 μM, respectively. The AUC data were moderately correlated inversely with IC_50_ data (Pearson’s *r* = -0.609; 2-tailed *p* = 0.027; N = 13) but not with the IC_25_ data (Pearson’s *r* = -0.268; 2-tailed *p* = 0.609; N = 13). The ratios of normalized AUC responses of **1** and **11** were lower than the ratio of **3** while the ratio of **12** was higher (Table 3, column 9). Likewise, the ratio of normalized AUC response of **6** was lower than that of **4** while the ratio of **10** was higher. As well, the ratio of normalized AUC response of **9** was lower than that of **8** while the ratio of **14** was higher. Evidently, monoglucosides **10**, **12** and **14** displayed higher AUC ratios compared to the respective archetypal isoflavone, while their 4΄-methoxy- (**1**), 3΄-methoxy- (**9**) and 3΄-hydroxy-derivatives (**6**) and the di-glucoside **11** displayed lower ratios. While seven isoflavonoids displayed an AUC ratio significantly different from the ratio of the respective archetypal isoflavone, only four isoflavonoids displayed an IC_50_ ratio significantly different from the IC_50_ of the respective archetypal isoflavone, possibly indicative of lower discriminative power of IC_50_-based comparisons relative to AUC-based ones.

**Table 3:** Induction of AlkP expression & ERE-dependent gene transcription.

Compound	Induction of ERE-dependent Luciferase Transcription (MCF-7:D5L cells			Induction of Alkaline Phosphatase Expression (Ishikawa cells)			Ratio of EC_50_	Ratio of AUC
	EC_25E2_ ^a^ (μM)	EC_50E2_ ^a^ (μM)	AUC ^b^ (%)	EC_25E2_ ^a^ (μM)	EC_50E2_ ^a^ (μM)	AUC ^b^ (%)	(AlkP / Lucif)^c^	(AlkP / Lucif)^d^
E2	3.60±0.72^#^	10.0±4.0^#^	100±9	9.83±0.44*	19.6±2.4^#^	111±17^#^	2.58±0.83	1.11±0.12
Genistein group								
**3**	0.03±0.01	0.09±0.03	100±17	0.03±0.01	0.05±0.02	124±19	0.63±0.02	1.24±0.12
**1**	0.16±0.09	0.30±0.03*	33±8*	0.19±0.01*	0.42±0.03*	26±2*	1.40±0.05*	0.79±0.04*
**11**	0.04±0.01	0.15±0.03	75±6	0.09±0.01*	0.16±0.02	66±5	1.12±0.09*	0.88±0.06*
**12**	0.05±0.01	0.13±0.03	69±7	0.05±0.01	0.07±0.01	110±10	0.57±0.04	1.59±0.10*
Isoprunetin group								
**4**	3.72±0.63	7.32±2.18	100±14	3.90±0.19	7.94±2.28	94±16	1.26±0.26	0.94±0.03
**6**	0.98±0.07*	1.57±0.24*	289±14*	1.60±0.05*	2.61±0.29	182±8*	1.69±0.10	0.63±0.01*
**7**	5.35±0.61	9.50±1.57	103±9	7.08±0.45*	11.8±2.5	76±15	1.23±0.17	0.74±0.13
**10**	6.50±1.10	29.8±5.2*	69±5	3.48±0.51	8.23±1.24	109±8	0.28±0.03*	1.58±0.12*
Daidzein group								
**5**	0.38±0.07	1.09±0.09	100±13	0.46±0.05	1.30±0.15	104±19	1.25±0.10	1.04±0.13
**2**	>30	>30	0.7±0.1*	>30	>30	0.5±0.2*	n-a	0.71±0.15
8-C-glucopyranosyl-genistein group								
**8**	0.42±0.05	1.38±0.59	100±21	0.66±0.06*	1.21±0.43	100±18	0.84±0.06	1.00±0.06
**9**	>30	>30	1.6±0.3*	>30	>30	1.1±0.1*	n-a	0.69±0.07*
**13**	2.20±0.18*	3.42±0.91	77±17	1.01±0.27	2.34±0.65	61±11	0.67±0.02*	0.79±0.13
**14**	3.57±0.58*	7.72±3.08	30±13*	3.72±0.31*	6.42±1.52*	46±10*	1.68±1.09	1.53±0.02*

^a^ compound concentration required to achieve 25% (IC_25_) or 50% (IC_50_) of the effect of 0.1 nM E2

^b^ %AUC was calculated by: [AUC_*n*_×100] / [AUC_*arch*_]; AUC_*arch*_ is the luciferase response of **3**, **4**, **5** or **8** up to 0.3, 10, 1.0 and 3.0 μM, respectively. AUC_*n*_ is the AlkP or luciferase response of isoflavone in group *n* up to the concentration used for the AUC_*arch*_ of group *n*

^c^ calculated by: [EC_50E2 AlkP_ / EC_50E2 luciferase_]

^d^ calculated by: [(AUC_AlkP_ / AUC_luciferase_)]

^#^ expressed in nM

*, *p<*0.05 *vs* the IC_25_, IC_50_, AUC of luciferase response of the respective archetypal compound (*t*-test)

E2 = estradiol

### Effects on osteoblastic and osteoclastic differentiation and on neuronal oxytosis

Since micromolar concentrations of isoflavonoids **3**, **11** and **12** displayed AlkP-inducing activity and ERα-dependent transcriptional activity comparable to physiological concentrations of estradiol but much higher compared to micromolar concentrations of the other isoflavonoids, we also compared their effects on the differentiation of MC3T3-E1 to osteoblasts using induction of AlkP and Alizarin red staining as markers of osteoblastic differentiation and mineralization, respectively. Incubation of MC3T3-E1 cells for 6 days with differentiation factors (DF: 10 mM beta-glucerophosphate, 50 μg/ml ascorbic acid plus test compound vehicle) or DF-free medium (plus test compound vehicle) resulted in 3.7-fold induction of AlkP expression in the presence of DF (Table 4, column 2). The DF-induced AlkP expression was increased in the presence of 1 nM estradiol or 1 μΜ **3, 11** or **12**, although the effect of **3** was not statistically significant. AlkP expression was not affected following treatment of DF-free MC3T3-E1 cells with test compounds. Similarly, incubation of MC3T3-E1 cells for 21 days with DF or DF-free medium resulted in 2.8-fold increase in Alizarin red staining in the presence of DF (Table 4, column 3). The DF-induced staining increased in the presence of estradiol, **11** and **12**, although the increase observed in the presence of the glucosides was not statistically significant. No increase in staining was observed following treatment of DF-free MC3T3-E1 cells with test compounds. We also determined the effect of estradiol, **3**, **11** and **12** on the differentiation of RAW 264.7 cells to osteoclasts, using induction of TRAP expression as differentiation marker. Incubation of RAW 264.7 cells for 3 days with 50 ng/ml RANKL (DF plus test compound vehicle) or RANKL-free medium (plus test compound vehicle) resulted in 2-fold induction of TRAP expression in the presence of RANKL (Table 4, column 4). The RANKL-induced increase in TRAP expression was not affected by estradiol, **3**, **11** or **12**. TRAP expression of RANKL-free RAW 264.7 cells was not affected following treatment with test compounds.

**Table 4:** Effects on osteoblastic and osteoclastic differentiation.

Treatment	Differentiation of MC3T3-E1 cells		Differentiation of RAW 267.4 cells
	AlkP expression (%)^a^	Alizarin red staining (%)^a^	TRAP expression (%)^a^
No DF + vehicle	27±4*	36±1*	51±3*
DF + vehicle	100±3	100±8	100±7
DF + E2 (1 nM)	118±2*	134±7*	105±3
DF + **3** (1 μM)	116±7	n-d	106±11
DF + **11** (1 μM)	120±6*	120±5	112±9
DF + **12** (1 μM)	125±8*	137±14	110±5

^a^ Effects in the presence of DF plus vehicle were set equal to 100

* *p*<0.05 vs DF + vehicle (ANOVA)

n-d, not determined; AlkP = alkaline phosphatase; DF = differentiation factors; TRAP = tartrate-resistant acid phosphatase

**Fig 6:**
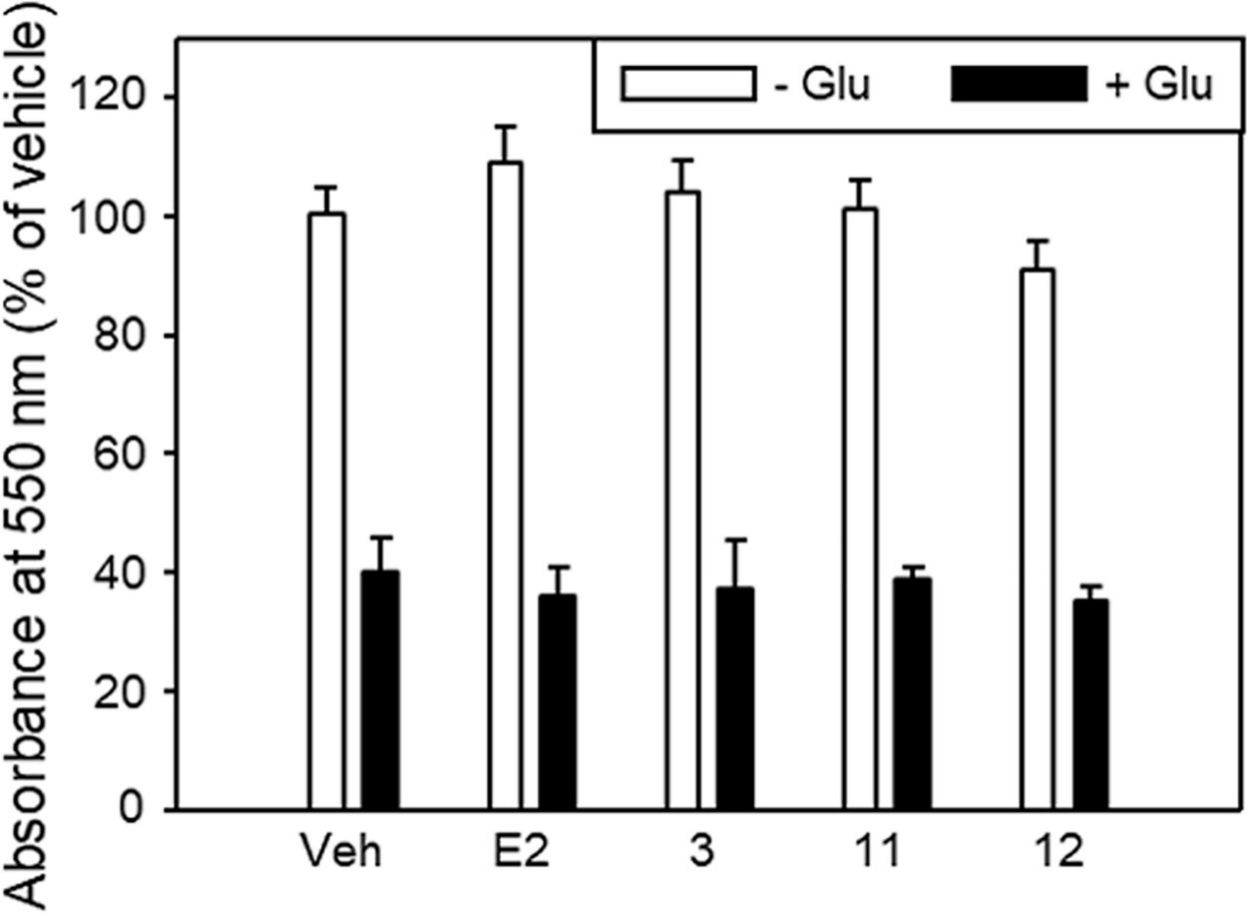
Viability of HT22 neuronal cells in the absence and presence of glutamate. HT22 cells were exposed to estradiol (E2, 1 nM), isoflavonoids (1 μM of **3**, **11** or **12**) or vehicle (Veh, 0.1% DMSO) and then challenged with 5 mM glutamate for 24 h prior to assessing the number of viable cells relative to that of vehicle-treated not challenged cells using MTT. Data are mean±SEM of three independent experiments in triplicate. Numbers of isoflavonoid-, E2- or vehicle-treated cells were similar (*p*>0.05, ANOVA) in the absence as well as the presence of glutamate.

HT22 cells are known to undergo oxidative stress-induced cell death (oxytosis) within 18–24 h following exposure to 5 mM glutamate. We therefore sought to determine whether estradiol, **3**, **11** or **12** could affect the viability of HT22 hippocampal neurons under normal growth conditions or following exposure to glutamate. Fig 6 shows that under normal growth conditions, the viability of HT22 cells growing in the presence of 1 nM estradiol or 1 μΜ **3**, **11** or **12** was not different compared to that of vehicle-treated HT22 cells (set equal to 100%; *p*>0.05, *t*-test). Treatment with glutamate reduced cell viability to ≤40% of that of glutamate-free cells, whether the treatment was carried out in the presence of vehicle, 1 nM estradiol or 1 μM **3**, **11** or **12**.

### Effects on TNFα mRNA expression of RAW 264.7 cells

**Fig 7:**
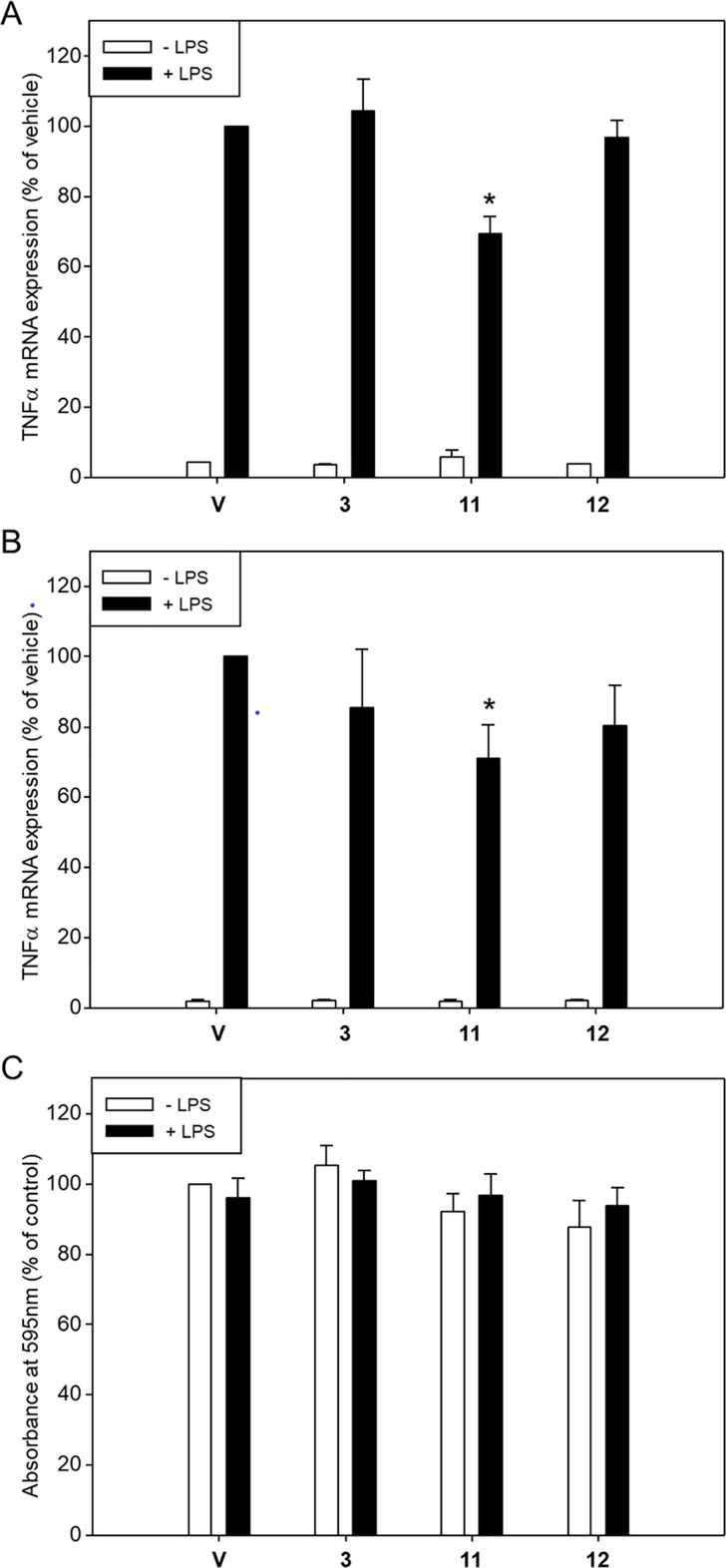
Effect of isoflavonoids on basal and LPS-induced TNFα mRNA expression in RAW 264.7 cells. Expression of TNFα mRNA in estrogen-free RAW 264.7 cells pre-treated for (A) 20 min or (B) 24 h with test compounds (3 μΜ) or vehicle (0.3% DMSO) prior to stimulation of the cells with LPS (100ng/ml) for 1h. Values are expressed as % of TNFα mRNA expression following treatment of vehicle pre-treated cells with LPS. (C) Relative numbers of RAW 264.7 cells pre-treated for 24 h with test compounds or vehicle and exposed or not to LPS, as assessed using crystal violet. Absorbance at 550 nm of vehicle pre-treated cells not exposed to LPS was set equal to 100. Values are mean±SEM of at least three independent experiments carried out in triplicate. *, *p*<0.05 *vs* TNFα mRNA levels in vehicle pre-treated LPS-treated cells (ANOVA).

The mouse macrophage cell line RAW 264.7 is known to express ERα and ERβ. Since the ER-dependent activities of **3**, **11** and **12** were much higher compared to the activities of the other isoflavonoids, we tested the ability of these three isoflavonoids to modulate transcription of the gene of pro-inflammatory cytokine TNFα in estrogen-free RAW 264.7 cells following 1-h stimulation of the cells with lipopolysaccharide (LPS). Pre-treatment of the cells with **3**, **11**, **12** or vehicle for 20 min prior to stimulation with LPS resulted in ~30-fold induction of TNFα mRNA expression in the presence of vehicle and in 30% reduction of this induction in the presence of **11** (Fig 7A). Pre-treatment of the cells with the test compounds for 24 h prior to the stimulation resulted in ~50-fold induction of TNFα mRNA expression in the presence of vehicle and in 15, 29 or 20% reduction of this induction in the presence of **3**, **11** or **12**, respectively, with the effect of **11** being statistically significant (Fig 7B). None of the isoflavonoids could affect TNFα mRNA expression in the absence of LPS. In addition, none of the isoflavonoids affected cell viability whether in the absence or presence of LPS (Fig 7C).

## Discussion

In an ongoing search for phytoestrogens from Mediterranean flora we found that the methanolic extract of the aerial parts of *Genista halacsyi* induced AlkP in Ishikawa cells. Induction of AlkP activity in Ishikawa cells is considered as a reliable measure of estrogenic activity [45]. This was not unexpected given that isoflavonoids with estrogen-like activity reportedly feature among the secondary metabolites of *Genista* species [1]. However, the phytochemical composition of *Genista halacsyi* had not yet been studied, and conventional chromatographic techniques had mainly been used for the isolation of compounds from plants belonging to this genus. We therefore exploited the advantages of FCPC (low risk of sample degradation, reversible adsorption of ingredients and low organic solvent consumption) in order to fractionate the extract. Several two-phase solvent systems have been reported for the chromatographic isolation of isoflavonoids from various natural sources [28]. Using a biphasic system consisting of EtOAc, EtOH and H_2_O we succeeded in fractionating the methanolic extract of *G*. *halacsyi* rapidly as well as effectively, and in isolating 14 isoflavonoids in pure form and in substantial amounts.

Assessment of RBAα and RBAβ of the isolated isoflavonoids revealed that **3** displayed ER-binding affinities and ERβ selectivity considerably higher than those of the other isoflavonoids. It has been reported that ERα and ERβ bind compounds with OH groups at a distance of 9.7–12.3 Å, through hydrogen bond formation with Glu353/Arg394 and His524 of ERα and Glu305/Arg346 and His475 of ERβ; that the ERβ selectivity of **3** likely depends predominantly on van der Waals contacts between its A and C rings and Ile373 and Met336 of ERβ, respectively; that the 5-OH and the keto group of the flavone part of **3** likely participate in intramolecular hydrogen-bond formation that gives rise to a planar 3-ring flavone system; and that the expanded planar structure of **3** in conjunction with the smaller ligand-binding pocket of ERβ compared to ERα likely provide for tighter packing of ERβ binding pocket residues around the flavone ring system of **3**, and hence for pronounced ERβ affinity and selectivity [53–55]. It is therefore likely that the smaller planar structure of **5** compared to **3**, due to the absence of a 5-OH in the former, reduces the number of ER pocket residues that could pack around the ligand and hence the ER-binding affinity and selectivity of **5** compared to **3**. It is also likely that introduction of bulkiness by methylation of 5-OH or by addition of a C*-*8 glucose moiety to generate **4** or **8**, respectively, prevents proper alignment for hydrogen bond formation through 4΄-OH and 7-OH, thus reducing their affinity and selectivity compared to **3**. Notably, **6** and **13** displayed higher RBAα compared to **4** and **8**, respectively, likely reflecting a comparatively higher hydrogen bonding ability of the 4΄-OH of **6** and **13**, due to the high electron donor potential of their 3΄-methoxy group. Similarly, the electron donor potential of 3΄-OH may account for the higher RBAα of **7** and **9** compared to **4** and **8**, respectively. Finally, as expected, **1**, **10**, **11**, **12** and **14**, which carry modified 4΄-OH and/or 7-OH groups, displayed considerably weaker RBA compared to **3**, **4** and **8**. Although **11** had RBA<0.01, **12** and **14** had measurable RBA for both ER subtypes. On **11**, the 7-OH and 4’-OH are substituted, while on **12** and **14,** 4’-OH and 7-OH, respectively, are free for hydrogen bond interaction. Although **12** was able to bind to ERα with very low affinity, it displayed estrogen-like activities much higher than excepted based on its RBAα, suggesting that these activities were due to hydrolytic release of the aglycone. Induced fit docking failed to predict binding mode for compound **14.** Probably, the binding cavity of 2P15 is not large enough to accommodate compound **14** and more residues should be shifted for binding. It is possible that **14** may have suffered limited hydrolysis at position 4’ during its isolation.

It is widely assumed that, although isoflavones display much lower ER-binding affinity compared to estradiol, their estrogen-like activity in vivo can match that of the hormone, given that their concentration in the circulation can reach levels up to 1 μΜ (i.e. ~10,000-fold higher than postmenopausal levels of the hormone) [13–15,56]. We showed that 0.1 nM estradiol displayed full AlkP-inducing, proliferative and ERα-transcriptional agonisms at concentrations ≥ 0.1 nM and this was the case also with **3**, **11** and **12** at concentrations ≥ 0.3 μM. We also showed that all isoflavonoid agonisms were inhibited by ICI182,780, suggestive of ER-dependent responses. In addition, we showed that AlkP-inducing agonisms were more strongly correlated with transcriptional agonisms through ERα than through ERβ. This correlation bias was clearly reflected in that e.g. **4** and **6** at 1 μΜ displayed partial and full, respectively, ERβ-dependent transcriptional agonisms, but marginal and weak, respectively, AlkP-inducing agonisms and ERα-dependent transcriptional agonisms. Thus, it appears that, although both ER subtypes are involved in regulating AlkP expression [51], the regulation is predominantly driven by ERα, presumably due to the dominant role that the latter reportedly plays in heterodimers with ERβ [9]. Moreover, we showed that none of the isoflavonoids displayed ER antagonist activity at 1 μΜ, suggesting that the substituents introduced in the archetypal structures failed to give rise to entities with antiestrogen-like and/or antiproliferative activity. However, this was reportedly not the case with some alkyl substitutions of the 7-OH of daidzein [57].

While the archetypal isoflavones **3**, **5** and **8** displayed higher activities compared to the other isoflavonoids of the respective group, **6** displayed higher activities compared to **4**, in accordance with their RBAα. However, the RBAα of **7**, **9** and **13** could not account for their lower activities compared to **4** and **8**, possibly reflecting variations in the ability of isoflavone-bound ERα to recruit transcriptional coactivators and/or to variations in cell uptake, metabolism and efflux of the aglycones. It has been reported that differential cofactor recruitment by phytoestrogen-bound ER may cause induction potencies to deviate considerably from predictions based solely on RBA [6]. In addition, it has been reported that the cellular availability of o-diphenolic compounds such as **7** and **9** could be drastically reduced by oxidation to ortho-quinones and by phase II metabolism to rapidly excreted sulfonate and glucuronide esters [13,14,58,59].

Unlike aglycones, which are more or less readily transported across plasma membranes, O-glucosides need processing by β-glucosidases before transport [11,12]. Deglycosylation by intestinal β-glucosidases is critically involved in the uptake of O-glucosides in humans and likely accounts for the significant variations observed in aglycone bioavailability [10,11,12]. O-glucosides **11** and **12**, in particular, displayed fairly similar AlkP-inducing and ERα-transcriptional activities compared to **3**. The presumptive beta-glucosidase activity that released **3** from **11** and **12**, is unlikely to have originated from the culture medium or from dead cells, given that cell viability was >95%, and serum was heat-inactivated for 1 h [60]. Most likely, this activity was secreted to the medium by living cells and/or was located in their plasma membrane [61]. In contrast to **11** and **12**, O-monoglucoside **14** displayed lower AlkP-inducing and ERα-transcriptional activities compared to **8**, implying that release of the 4΄-O-linked glucose moiety of **14** could be impeded by its 8-C-linked glucose moiety.

Although potency is widely used to report data on *in vitro* biological evaluation of test compounds, an AUC metric fitted over the entire range of comparatively relevant test compound concentrations combines both efficacy and potency and is considered as a more reliable measure of bioactivity [62,63]. The AUC of AlkP and ERα-transcriptional responses of isoflavonoids of a group were normalized using the AUC of the transcriptional response of the respective archetypal isoflavone. The relative AUC ratios revealed that the 3΄-OH and 3΄-methoxy derivatives **6**, **7**, **9** and **13** displayed AlkP responses ~30% lower than ERα-transcriptional responses, possibly reflecting a lower availability of these derivatives compared to the archetypal isoflavones that predominantly affected the AlkP response. Similarly, the lower relative AUC ratio of **11** compared to **3** may indicate a lower availability of di-glucoside-derived aglycone compared to the free one that disfavored the AlkP response. In contrast, monoglucosides **10**, **12** and **14** displayed AlkP responses ~40% higher than ERα-transcriptional responses, possibly indicating a relative availability of monoglucoside-derived aglycones compared to the free ones that favored the AlkP response. Indeed, comparison of the rank order of AlkP-induction efficacies of **3**, **11** and **12** at 0.3 μM (**3** = **12**>**11**; Fig 5A) to the rank order of the respective transcriptional efficacies (**3**>**12** = **11**; Fig 4A) could partly account for the finding that **11** and **12** displayed lower and higher, respectively, AUC ratio of AlkP response to ERα-transcriptional response compared to the aglycone.

Differentiation of MC3T3-E1 cells to mineralizing osteoblasts reportedly recapitulates *in vitro* many estrogen and ERα-dependent effects that promote osteoblastic differentiation *in vivo* and therefore has been extensively used to screen for phytoestrogens with osteoprotective activity [37,38]. We showed that estradiol, **11** and **12** promoted differentiation of MC3T3-E1 cells to osteoblasts, while **3** was not convincingly effective in this respect. In addition, estradiol and **12** promoted mineralization of differentiated MC3T3-E1 cells, while **11** was not convincingly effective in this respect. Part of these findings is in accordance with previous reports on effects of estradiol and genistein on the osteoblastic differentiation and mineralization of MC3T3-E1 cells [64]. In addition, they show that the O-glucosides of genistein stimulate osteoclastic differentiation of MC3T3-E1 cells as effectively as estradiol.

We also looked for effects of estradiol, **3**, **11** and **12** on osteoclast differentiation of RAW 264.7 cells following treatment with RANKL (50 ng/ml) using induction of TRAP expression as differentiation marker. We observed no change of the basal or the RANKL-induced TRAP activity in the presence of 1 nΜ estradiol or 1 μM of **3**, **11** or **12**. These findings are in accordance with previous reports that estradiol and genistein can inhibit osteoclastic differentiation of RAW 264.7 cells at concentrations ≥10 μM [65,66]. Similarly, it has been reported that the inhibition of differentiation of RAW 264.7 cells to osteoclasts by resveratrol and other flavonoids depends predominantly on their antioxidant activity and that the inhibition is appreciable provided these compounds are tested at high concentrations [65,67]. Thus, it appears that estradiol, **3**, **11** and **12** at physiologically and/or pharmacologically relevant concentrations are unable to inhibit osteoclastic differentiation of RAW 264.7 cells. In line with this notion, it has been reported that stimulation of osteoblastic differentiation of MC3T3-E1 cells by soybean extract or silibinin (a flavonolignan) endows the culture medium with factors capable of inhibiting osteoclastic differentiation of RAW 264.7 [68,69], implying that isoflavones inhibit differentiation of monocytes to osteoclasts indirectly.

Using HT22 cells, a ER-expressing hippocampal cell line used for screening chemical libraries for neuroprotective compounds [42,43,70,71], we showed that estradiol at 1 nM and **3**, **11** or **12** at 1 μM failed to display neuroprotective activity. Estradiol is reportedly able to protect glutamate-challenged HT22 cells from oxytosis in an ER-dependent manner, although at supraphysiological concentrations (IC50>800 nM) and in an ICI182,780-independent manner at high physiological concentrations (50 nM) [71], implying that the involvement of ER in estrogen-dependent oxytosis prevention in HT22 cells is minor, if not obsolete. Others have also reported that even 1 μM estradiol or 2.5 μM genistein are unable to prevent oxytosis of glutamate-challenged HT22 cells [72,73]. However, given that the half maximal concentration for HT22 oxytosis inhibition by renowned antioxidants such as resveratrol and fisetin is ≥3 μM [43,70], one cannot exclude that **3**, **11** and **12** may inhibit oxytosis at higher (yet not pharmacologically relevant) concentrations, as already shown for estradiol [72].

LPS is predominantly involved in stimulating TNFα mRNA expression and production of this cytokine following infection of monocytes. We have shown that compared to **3** which was ineffective and to **12** which was marginally effective, **11** displayed a ~30% suppression of LPS-stimulated TNFα mRNA expression in estrogen-free RAW 264.7 cells pre-incubated with 3 μM isoflavonoid prior to the stimulation. The higher efficacy of the 7,4΄-di-O-glucoside of genistein to prevent LPS-induced TNFα transcription compared to the respective 7-O-mono-glucoside and the aglycone may reflect a higher availability of di-glucoside-derived aglycone compared to the mono-glucoside-derived one and to the free aglycone. However, others have reported that pre-incubation with low micromolar concentrations of genistein is capable of causing 30–40% inhibition of TNFα gene transcription in RAW 264.7 cells [74]. These findings indicate that the extent to which genistein can moderate LPS-induced TNFα mRNA expression of RAW 264.7 cells may depend on cell culture conditions and/or on using specific clones of RAW264.7 cells [41]. Although the mouse macrophage cell line RAW 264.7 is known to express ERα and ERβ, estradiol was reportedly unable to suppress TNFα mRNA expression and release of this cytokine following stimulation of estrogen-free RAW 264.7 cells with LPS [75]. Others have reported, however, that pretreatment of estrogen-free RAW 264.7 cells with 1–10 μM equol reportedly resulted in dose-dependent suppression of LPS-induced TNFα mRNA expression and cytokine release in an ER-independent manner [76].

The genitourinary syndrome of menopause (GSM) is known to affect more than 50% of menopausal women, especially breast cancer survivors, to whom even low-dose vaginal estrogen may be unacceptable due to concerns over possible breast or endometrial cancer risk [77,78]. While orally administered isoflavones are extensively metabolized and rapidly conjugated and excreted, topically or parenterally administered isoflavones are found in the circulation in unconjugated form in appreciable amounts [12]. Accordingly, topically administered isoflavones may reportedly relieve GSM as effectively as vaginal estrogen [79]. The present data on the estrogen-like activity of isoflavonoids may be taken to suggest that formulations rich in O*-*glucosides of genistein could be useful for topical treatment of GSM.

## Conclusion

We have determined the estrogen-like activities of 14 isoflavonoids isolated from the aerial parts of *Genista halacsyi* using FCPC. We have shown that the estrogen-like activities of O-glucosides were similar to those of the respective aglycones, while the activities of C-glucosides were lower. We have also shown that low micromolar concentrations of genistein and its 7-O-mono- and 7,4΄-di-O-glucosides were at least as active as physiological concentrations of estradiol. Our findings could be taken to indicate that topical use of formulations rich in O*-*glucosides of genistein could substitute for low-dose vaginal estrogen for the treatment of GMS. However, further studies using the ovariectomized mouse model of menopause are required in order to determine whether topical application of genistein and/or its glucosides may have GSM chemopreventive and/or therapeutic potential.

## Supporting information

S1 File Biphasic solvent systems tested for the FCPC analysis; Spectroscopic data for compounds 1–14; LogP values of compounds 1–14 as predicted from QikProt software. (PDF)
