# Supplementary material for: Biological evaluation of isoflavonoids from Genista halacsyi using estrogen-target cells: Activities of glucosides compared to aglycones
Source: PLoS One. 2019 Jan 8;14(1):e0210247. doi: 10.1371/journal.pone.0210247 (PMC6324813; doi:10.1371/journal.pone.0210247)
Supplement: S1 File — (PDF) [file pone.0210247.s001.pdf]

Supporting Information

Biological evaluation of isoflavonoids from *Genista halacsyi* using estrogen-target cells: Activities of glucosides compared to aglycones

Nikolas Fokialakis[1¶*], Xanthippi Alexi[2¶], Nektarios Aligiannis[1], Athina Boulaka[2], Aggeliki K. Meligova[2], George Lambrinidis[3], Eleftherios Kalpoutzakis[1], Harris Pratsinis[3], Antigoni Cheilari[1], Dimitra J. Mitsiou[2], Sofia Mitakou[1], Michael N. Alexis[2*]

[1] Department of Pharmacognosy and Natural Products Chemistry, Faculty of Pharmacy, National and Kapodistrian University of Athens, Athens, Greece

[2] Institute of Biology, Medicinal Chemistry and Biotechnology, National Hellenic Research Foundation, Athens, Greece

[3] Division of Pharmaceutical Chemistry, Faculty of Pharmacy, National and Kapodistrian University of Athens, Athens, Greece

[4] Laboratory of Cell Proliferation & Ageing, Institute of Biosciences & Applications, NCSR "Demokritos", Athens, Greece

[¶] These authors have contributed equally to this work

***Email**: mnalexis@eie.gr (MNA); fokialakis@pharm.uoa.gr (NF)

Table of Contents Page

Table A: Biphasic solvent systems tested for the FCPC analysis...3

Table B: Spectroscopic data for compound **1**..4

Table C: Spectroscopic data for compound **2.**..5

Table D: Spectroscopic data for compound **3.**..6

Table E: Spectroscopic data for compound **4.**..7

Table F: Spectroscopic data for compound **5**..8

Table G: Spectroscopic data for compound **6**..9

Table H: Spectroscopic data for compound **7**..10

Table I: Spectroscopic data for compound **8**...11

Table J: Spectroscopic data for compound **9**...12

Table K: Spectroscopic data for compound **10**...13

Table L: Spectroscopic data for compound **11**...14

Table M: Spectroscopic data for compound **12**...15

Table N: Spectroscopic data for compound **13**..16

Table O: Spectroscopic data for compound **14**..17

Table P: LogP values (QPlogP) as predicted from QikProt software..................................18

Table A: Biphasic solvent systems tested for the FCPC analysis

No	Solvents system	Evaluation results
1	Hexane/EtOAc/MeOH/H_2O 2/8/2/8	Unsatisfactory distribution of ingredients / Inappropriate system
2	Hexane/EtOAc/MeOH/H_2O 2/4/2/4	Unsatisfactory distribution of ingredients / Inappropriate system
3	Hexane/EtOAc/MeOH/H_2O 1/6/1/6	Unsatisfactory distribution of ingredients / Inappropriate system
4	Hexane/EtOAc/MeOH/H_2O 8/6/8/6	Unsatisfactory distribution of ingredients / Inappropriate system
5	Hexane/EtOAc/MeOH/H_2O 1/5/1/5	Unsatisfactory distribution of ingredients / Inappropriate system
6	Hexane/EtOAc/MeOH/H_2O 1/3/1/3	Unsatisfactory distribution of ingredients / Inappropriate system
7	Hexane/EtOAc/MeOH/H_2O 1/9/1/9	Unsatisfactory distribution of ingredients / Inappropriate system
8	EtOAc/MeOH/H_2O 10/1/10	Satisfactory distribution of ingredients / Appropriate system
9	$CHCl_3$/MeOH/H_2O 8/10/5	Emulsion formation / Inappropriate system
10	CH_2Cl_2/MeOH/H_2O 4/3/2	Unsatisfactory distribution of ingredients / Inappropriate system
11	$CHCl_3$/MeOH/H_2O 4/3/2	Emulsion formation / Inappropriate system
12	$CHCl_3$/MeOH/n-BuOH/H_2O 7/6/3/4	Unsatisfactory distribution of ingredients / Inappropriate system
13	$CHCl_3$/MeOH/n-BuOH/H2O 6/5/3/6	Emulsion formation / Inappropriate system
14	$CHCl_3$/MeOH/n-BuOH/H2O 7/4/4/5	Emulsion formation / Inappropriate system

Table B: Spectroscopic data for compound **1** (Biochanin A)

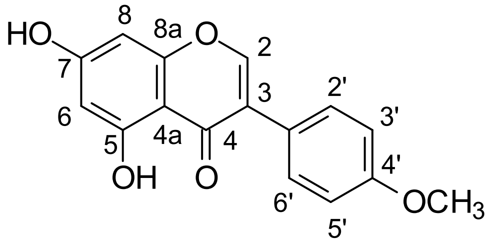

$C_{16}H_{12}O_5$
284.26

- ^1H NMR (DMSO) - ^{13}C NMR (DMSO)

	^1H	^{13}C
1	-	-
2	8.36 (1H, s)	154.2
3	-	122.9
4	-	180.1
4a	-	104.5
5	-	162.0
6	6.22 (1H, d, J = 2.4 Hz)	93.7
7	-	157.6
8	6.38 (1H, d, J = 2.4 Hz)	98.9
8a	-	159.1
1'	-	121.9
2' / 6'	7.48 (2H,d, J = 8.7 Hz)	130.1
4'	-	164.3
3'/5'	6.99 (2H,d, J = 8.7 Hz)	113.7
4'- OCH_3	3.77 (3H, s)	55.1

Table C: Spectroscopic data for compound **2** (8-Methoxyformononetin)

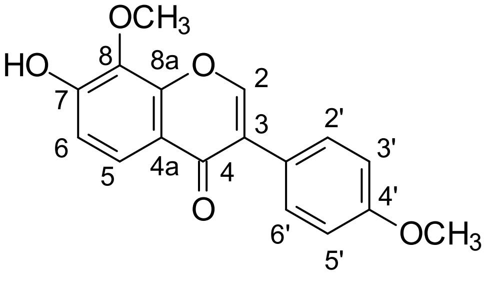

$C_{17}H_{14}O_5$
298.29

- ^1H NMR ($CDCl_3$) - ^{13}C NMR ($CDCl_3$)

	^1H	^{13}C
1	-	-
2	7.98 (1H, s)	152.1
3	-	124.2
4	-	176.8
4a	-	119.0
5	7.96 (1H,d, *J* = 8.0 Hz)	121.1
6	7.03 (1H, d, *J* = 8.0 Hz)	110.7
7	-	154.4
8	-	134.0
8-OCH_3	4.10 (3H, s)	56.6
8a	-	151.4
1’	-	123.8
2’/6’	7.48 (2H,d, *J* = 8.0 Hz)	131.8
4’	-	160.0
3’/5’	6.87 (2H,d, *J* = 8.0 Hz)	114.3
4’-OCH_3	3.78 (3H, s)	56.0

Table D: Spectroscopic data for compound **3** (Genistein)

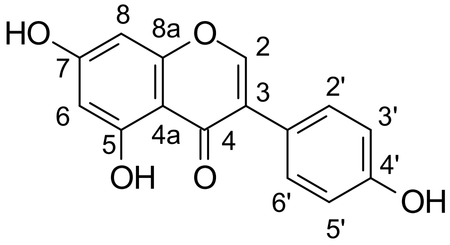

$C_{15}H_{10}O_5$
270.24

- ^{1}H NMR (DMSO) - ^{13}C NMR (DMSO)

	^{1}H	^{13}C
1	-	-
2	8.31 (1H, s)	153.8
3	-	122.2
4	-	180.1
4a	-	104.4
5	-	162.0
6	6.22 (1H, d, *J* = 2.0 Hz)	98.9
7	-	164.3
8	6.38 (1H, d, *J* = 2.0 Hz)	93.6
8a	-	157.5
1’	-	121.1
2’/6’	7.37 (2H,d, *J* = 8.0 Hz)	130.0
4’		157.4
3’/5’	6.81 (2H,d, *J* = 8.0 Hz)	115.0

Table E: Spectroscopic data for compound **4** (Isoprunetin)

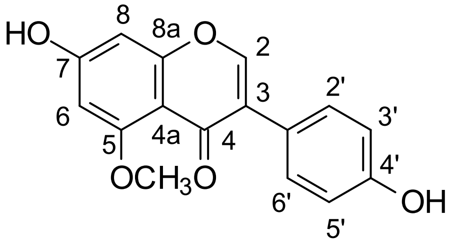

$C_{16}H_{12}O_5$
284.26

- ^1H NMR (DMSO) - ^{13}C NMR (CD_3OD)

	^1H	^{13}C
1	-	-
2	8.01 (1H, s)	152.9
3	-	127.4
4	-	175.8
4a	-	109.7
5	-	163.5
5-OCH_3	3.78 (1H, s)	56.8
6	6.35 (1H, s)	96.5
7	-	165.2
8	6.37 (1H, s)	97.8
8a	-	161.9
1’	-	125.0
2’/6’	7.29 (2H,d, *J* = 8.5 Hz)	132.0
4’	-	159.0
3’/5’	6.76 (2H,d, *J* = 8.5 Hz)	116.5

Table F: Spectroscopic data for compound **5** (Daidzein)

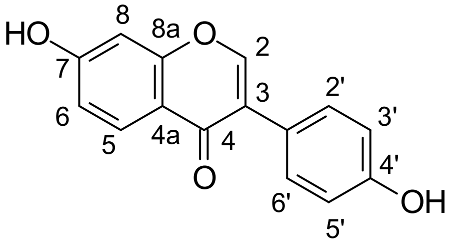

$C_{15}H_{10}O_4$
254.24

- ^1H NMR (DMSO) - ^{13}C NMR (DMSO)

	^1H	^{13}C
1	-	-
2	8.22 (1H, s)	152.9
3	-	123.5
4	-	175.1
4a	-	116.7
5	7.90 (1H, d, *J* = 8.0 Hz)	127.3
6	6.89 (1H, dd, *J* = 8.0 / 1.5 Hz)	115.9
7	-	163.6
8	6.81 (1H, d, *J* = 1.5 Hz)	102.2
8a	-	158.3
1'	-	122.4
2'/6'	7.32 (2H,d, *J* = 8.0 Hz)	130.1
4'		158.1
3'/5'	6.78 (2H,d, *J* = 8.0 Hz)	115.8

Table G: Spectroscopic data for compound **6** (3´-methoxyisoprunetin or Gerontoisoflavone A)

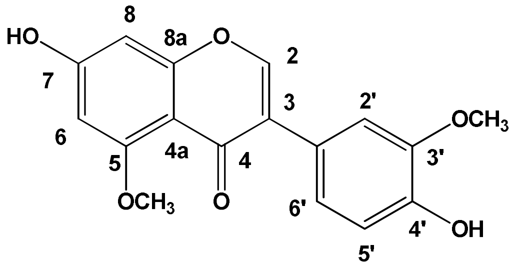

$C_{17}H_{14}O_6$
314,29

- ^{1}H NMR (CDCl3) ^{13}C NMR (CD_3OD)

	^{1}H	^{13}C
1	-	-
2	8.00 (1H, s)	152.5
3	-	126.7
4	-	174.9
4a	-	109.4
5	-	163.7
5-OCH_3	3.88 (1H, s)	56.6
6	6.43 (1H, s)	96.8
7	-	165.3
8	6.43 (1H, s)	97.4
8a	-	161.7
1’	-	124.5
2’	7.16 (2H,d, J = 2.0 Hz)	114.1
3’		147.9
3’-OCH_3	3.86 (1H, s)	56.4
4’	-	145.8
5’	6.85 (2H,d, J = 8.3 Hz)	115.9
6’	6.94 (2H,d, J = 8.3, 2.0 Hz)	121.8

Table H: Spectroscopic data for compound **7** (5-*O*-methylorobol)

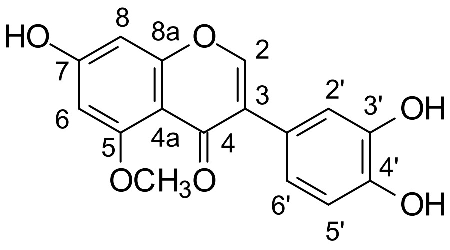

$C_{16}H_{12}O_6$
300.26

- ^{1}H NMR (CD_3OD + C_6D_6) - ^{13}C NMR ($(CD_3)_2CO$)

	^{1}H	^{13}C
1	-	-
2	7.75 (1H,s)	150.1
3	-	125.1
4	-	174.1
4a	-	109.8
5	-	163.3
5-OCH_3	3.93 (3H, s)	57.0
6	6.39 (1H, d, *J* = 2.0 Hz)	96.3
7	-	163.3
8	6.42 (1H, d, *J* = 2.0Hz)	96.3
8a	-	159.9
1'	-	124.2
2'	7.02 (1H,d, *J* = 1.8 Hz)	117.3
3'	-	145.7
4'	-	145.5
5'	6.83 (1H,d, *J* = 8.0Hz)	123.0
6'	6.79 (1H,dd, *J* = 8.0, 1.8 Hz)	120.0

Table I: Spectroscopic data for compound **8** (8-*C*-glucopyranosylgenistein)

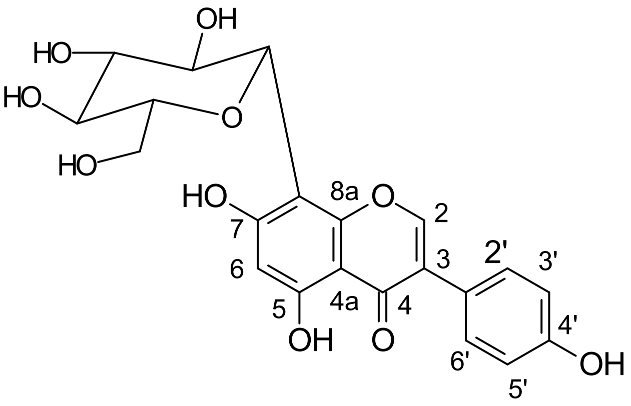

$C_{21}H_{20}O_{10}$
432.38

- ^{1}H NMR (CD_3OD) - ^{13}C NMR (CD_3OD)

	^{1}H	^{13}C
2	8.11 (1H,s)	154.6
3	-	124.0
4	-	182.2
4a	-	106.3
5	-	163.1
6	6.29 (1H, s)	100.3
7	-	164.4
8	-	104.1
8a	-	158.4
1’	-	122.9
2’/6’	7.37 (2H,d, *J* = 8.6 Hz)	131.1
4’	-	158.4
3’/5’	6.85 (2H,d, *J* = 8.6 Hz)	116.1
1’’	4.96 (1H, d, *J* = 9.8 Hz)	75.2
2’’	4.14 (1H, t, *J* = 9.8 Hz)	72.7
3’’	3.56-3.42 (1H, *)	79.9
4’’	3.56-3.42 (1H, *)	71.6
5’’	3.56-3.42 (1H, *)	82.3
6’’	3.91 (1H, brd, *J* = 11.7 Hz) / 3.75 (1H, dd, *J* = 11.7, 4.5 Hz)	62.7

*Overlapped peaks

Table J: Spectroscopic data for compound **9 (**8-*C*-glucopyranosyl-orobol)

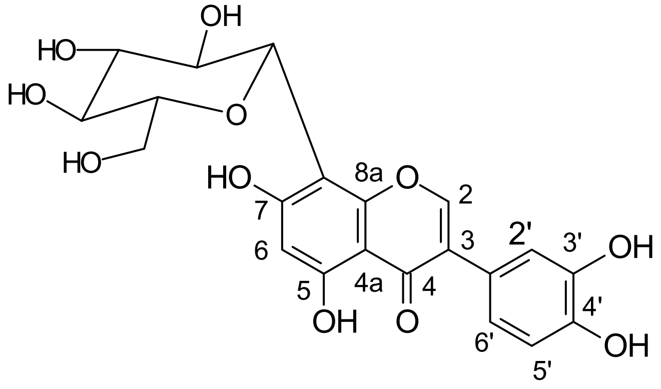

$C_{21}H_{20}O_{11}$
448.38

- ^{1}H NMR (CD_3OD) - ^{13}C NMR (CD_3OD)

	^{1}H	^{13}C
1	-	-
2	8.10 (1H,s)	153.6
3	-	123.8
4	-	181.0
4a	-	105.1
5	-	162.4
6	6.30 (1H, s)	99.4
7	-	163.8
8	-	103.4
8a	-	156.3
1’	-	122.6
2’	7.04 (1H,d, *J* = 1.9 Hz)	116.4
3’	-	145.3
4’	-	145.8
5’	6.84 (1H,d, *J* = 8.1 Hz)	115.2
6’	6.86 (1H,dd, *J* = 8.1, 1.9 Hz)	121.2
1’’	4.96 (1H, d, *J* = 9.7 Hz)	74.7
2’’’	4.14 (1H, t, *J* = 9.8 Hz)	71.7
3’’	3.59-3.40 (1H, *)	79.5
4’’	3.59-3.40 (1H, *)	70.7
5’’	3.59-3.40 (1H, *)	82.5
6’’	3.91 (1H, brd, *J* = 12.2 Hz) / 3.75 (1H, dd, *J* = 12.2, 2.5 Hz)	62.2

*Overlapped peaks

Table K: Spectroscopic data for compound **10** (7-*O*-glucopyranosyl-isoprunetin)

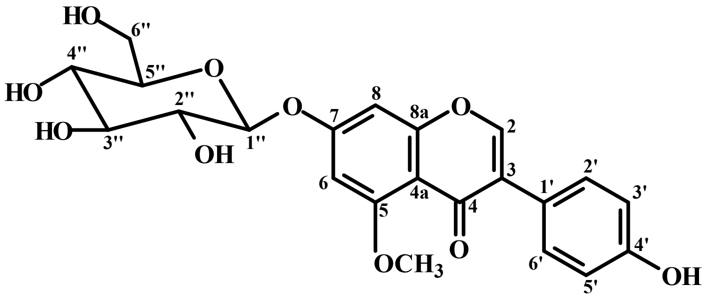

- ^{1}H NMR (CD_3OD) ^{13}C NMR (CD_3OD)

	^{1}H	^{13}C
1	-	-
2	7.96 (1H,s)	152.5
3	-	126.9
4	-	177.4
4a	-	111.1
5	-	162.1
5-CH_3O	3.93 (3H, s)	56.6
6	6.69 (1H, d, *J* = 2.4 Hz)	96.4
7	-	163.0
8	6.77 (1H, d, *J* = 2.4 Hz)	97.3
8a	-	160.4
1’	-	123.6
2’/6’	7.35 (2H,d, *J* = 8.8 Hz)	131.6
4’		158.1
3’/5’	6.84 (2H,d, *J* = 8.8 Hz)	115.9
1’’	5.06 (1H, d, *J* = 7.3 Hz)	101.6
2’’	3.59-3.41 (1H,*)	73.4
3’’	3.59-3.41 (1H,*)	78.7
4’’	3.59-3.41 (1H,*)	71.2
5’’	3.59-3.41 (1H,*)	78.7
6’’	3.95 (1H, brd, *J* = 12.2 Hz) / 3.73 (1H, dd, *J* = 12.2, 6.4 Hz)	62.6

Table L: Spectroscopic data for compound **11** (7,4´-di-*O*-glucopyranosylgenistein)

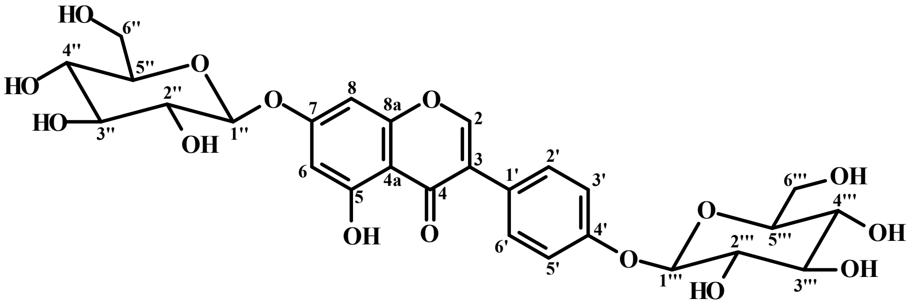

- ^{1}H NMR (CD_3OD) - ^{13}C NMR (CD_3OD)

	^{1}H	^{13}C
1	-	-
2	8.20 (1H,s)	155.3
3	-	121.9
4	-	182.0
4a	-	107.0
5	-	162.5
6	6.53 (1H, d, *J* = 2.0 Hz)	100.4
7	-	163.9
8	6.72 (1H, d, *J* = 2.0 Hz)	95.9
8a	-	158.1
1’	-	123.4
2’/6’	7.52 (2H,d, *J* = 8.3 Hz)	131.0
4’		159.6
3’/5’	7.19 (2H,d, *J* = 8.3 Hz)	117.8
1’’	5.07 (1H, d, *J* = 7.1 Hz)	101.9
2’’	3.96-3.38 (1H,*)	73.9
3’’	3.96-3.38 (1H,*)	78.6
4’’	3.96-3.38 (1H,*)	71.2
5’’	3.96-3.38 (1H,*)	78.2
6’’	3.96-3.38 (2H, *)	62.1
1’’’	4.98 (1H, *)	101.9
2’’’	3.96-3.38 (1H,*)	73.8
3’’’	3.96-3.38 (1H,*)	78.8
4’’’	3.96-3.38 (1H,*)	71.4
5’’’	3.96-3.38 (1H,*)	78.1
6’’’	3.96-3.38 (2H, *)	62.3

Table M: Spectroscopic data for compound **12** (7-*O*-β-D-glucopyranosyl-genistein)

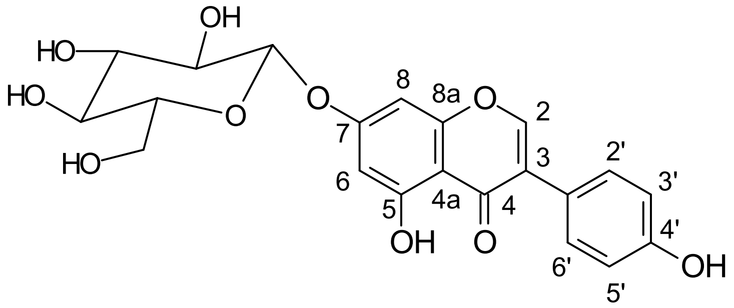

$C_{21}H_{20}O_{10}$
432.38

- ^{1}H NMR (DMSO) - ^{13}C NMR (DMSO)

	^{1}H	^{13}C
1	-	-
2	8.35 (1H,s)	155.4
3	-	121.9
4	-	182.0
4a	-	107.0
5	-	162.5
5-OH	12.93 (1H, s)	-
6	6.55 (1H, d, *J* = 2.2 Hz)	100.4
7	-	163.9
8	6.79 (1H, d, *J* = 2.2 Hz)	95.4
8a	-	158.1
1’	-	123.4
2’/6’	7.44 (2H,d, *J* = 8.7 Hz)	131.0
4’		158.3
3’/5’	6.91 (2H,d, *J* = 8.7 Hz)	115.9
4’-OH	9.95 (1H, s)	-
1’’	5.09 (1H, d, *J* = 7.1 Hz)	100.7
2’’	3.55-3.31 (1H,*)	73.9
3’’	3.55-3.31 (1H,*)	77.2
4’’	3.55-3.31 (1H,*)	70.4
5’’	3.55-3.31 (1H,*)	78.1
6’’	3.79 (1H, d, *J* = 12.1 Hz) / 3.55-3.31 (1H,*)	61.5

*Overlapped peaks

Table N: Spectroscopic data for compound **13 (**8-*C*-glucopyranosyl-3´-*O*-methylorobol)

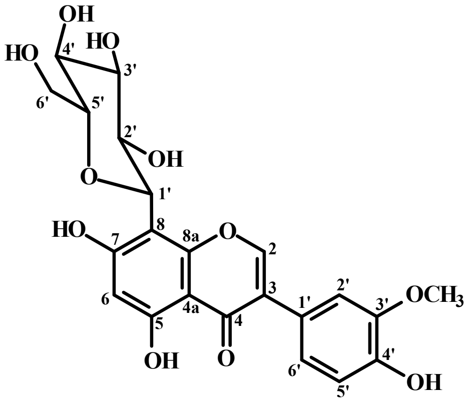

- ^{1}H NMR (CD_3OD) - ^{13}C NMR (CD_3OD)

	^{1}H	^{13}C
1	-	-
2	8.18 (1H,s)	153.8
3	-	123.7
4	-	181.6
4a	-	105.3
5	-	162.4
6	6.30 (1H, s)	99.6
7	-	163.7
8	-	103.5
8a	-	156.4
1’	-	122.5
2’	7.18 (1H,d, *J* = 1.9 Hz)	116.6
3’	-	145.6
3’-CH_3O	3.90 (3H, s)	56.9
4’	-	145.9
5’	6.87 (1H,d, *J* = 7.8 Hz)	115.4
6’	6.99 (1H,dd, *J* = 7.8, 2.0 Hz)	121.2
1’’	4.96 (1H, d, *J* = 9.8 Hz)	74.7
2’’’	4.13 (1H, t, *J* = 9.8 Hz)	71.8
3’’	3.59-3.40 (1H, *)	79.7
4’’	3.59-3.40 (1H, *)	70.8
5’’	3.59-3.40 (1H, *)	82.3
6’’	3.91 (1H, dd, *J* = 11.6, 2.0 Hz) / 3.75 (1H, dd, *J* = 11.6, 4.9 Hz)	62.4

*Overlapped peaks

Table O: Spectroscopic data for compound **14 (**8-*C*,4′-*O*-diglucopyranosyl-genistein)

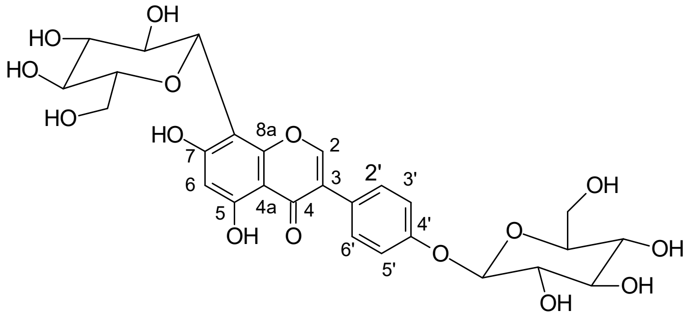

- ^{1}H NMR (DMSO) ^{13}C NMR (DMSO)

	^{1}H	^{13}C
1	-	-
2	8.38 (1H,s)	154.0
3	-	121.4
4	-	180.1
4a	-	104.2
5	-	160.8
6	6.21 (1H, s)	99.3
7	-	163.1
8	-	104.2
8a	-	157.1
1’	-	124.1
2’/6’	7.49 (2H,d, *J* = 9.0 Hz)	129.9
4’		156.4
3’/5’	7.08 (2H,d, *J* = 9.0 Hz)	115.9
1’’	4.90 (1H, d, *J* = 7.0 Hz)	100.2
2’’	3.81-3.31 (1H, *)	70.5
3’’	3.81-3.31 (1H, *)	78.7
4’’	3.81-3.31 (1H, *)	69.6
5’’	3.81-3.31 (1H, *)	81.5
6’’	3.81-3.31 (2H, *)	61.3
1’’’	4.85 (1H, d, *J* = 10.0 Hz)	73.1
2’’’	3.81-3.31 (1H, *)	72.7
3’’’	3.81-3.31 (1H, *)	79.9
4’’’	3.81-3.31 (1H, *)	71.6
5’’’	3.81-3.31 (1H, *)	82.3
6’’’	3.81-3.31 (2H, *)	62.1

Table P: LogP values (QPlogP) as predicted from QikProt software.

Compound	QPlogP
EST	3.989
1	2.522
2	2.746
3	1.725
4	1.965
5	1.820
6	2.090
7	1.299
8	-0.532
9	-1.157
10	-0.030
11	-2.292
12	-0.301
13	-0.528
14	-2.561
